# Inactivation of Chk2 and Mus81 Leads to Impaired Lymphocytes Development, Reduced Genomic Instability, and Suppression of Cancer

**DOI:** 10.1371/journal.pgen.1001385

**Published:** 2011-05-19

**Authors:** Samah El Ghamrasni, Ashwin Pamidi, Marie Jo Halaby, Miyuki Bohgaki, Renato Cardoso, Li Li, Shriram Venkatesan, Swaminathan Sethu, Atsushi Hirao, Tak W. Mak, Manoor Prakash Hande, Anne Hakem, Razqallah Hakem

**Affiliations:** 1 Ontario Cancer Institute, University Health Network and Department of Medical Biophysics, University of Toronto, Toronto, Canada; 2 Department of Physiology, Yong Loo Lin School of Medicine, National University of Singapore, Singapore, Singapore; 3 Division of Molecular Genetics, Center for Cancer and Stem Cell Research, Cancer Research Institute, Kanazawa University, Kanazawa, Ishikawa, Japan; 4 The Campbell Family Institute for Breast Cancer Research, Toronto, Canada; University of Washington, United States of America

## Abstract

Chk2 is an effector kinase important for the activation of cell cycle checkpoints, p53, and apoptosis in response to DNA damage. Mus81 is required for the restart of stalled replication forks and for genomic integrity. *Mus81^Δex3-4/Δex3-4^* mice have increased cancer susceptibility that is exacerbated by p53 inactivation. In this study, we demonstrate that Chk2 inactivation impairs the development of *Mus81^Δex3-4/Δex3-4^* lymphoid cells in a cell-autonomous manner. Importantly, in contrast to its predicted tumor suppressor function, loss of Chk2 promotes mitotic catastrophe and cell death, and it results in suppressed oncogenic transformation and tumor development in *Mus81^Δex3-4/Δex3-4^* background. Thus, our data indicate that an important role for Chk2 is maintaining lymphocyte development and that dual inactivation of Chk2 and Mus81 remarkably inhibits cancer.

## Author Summary

Failure to repair DNA damage has been associated with a number of human syndromes, neurodegenerative diseases, immunodeficiency, and cancer. In addition, radiotherapy and many cancer chemotherapeutic drugs induce DNA damage, thus allowing the killing of tumors. Recent data indicated Mus81's role in maintaining genomic integrity and suppressing cancer. Furthermore, inactivation of p53, the most frequently inactivated tumor suppressor in cancer, leads to synergistic tumorigenesis in *Mus81* mutant mice. As Chk2 is important for p53 activation, we have examined the effect of its inactivation on the phenotypes associated with Mus81 loss of function. We report that Chk2 is essential for the development of lymphoid cells deficient for Mus81. Chk2 inactivation increased spontaneous cell death of *Mus81* deficient cells and impaired the development of T and B-cell lineages. Chk2 inactivation also reduced the frequency of Mus81-deficient cells that carry elevated levels of spontaneous genomic instability. Importantly, inactivation of Chk2 protected *Mus81* mutant mice from developing spontaneous tumorigenesis. These data indicate potential therapeutic benefits for the inactivation of Chk2 and Mus81.

## Introduction

DNA damage response is a result of the coordinated actions of DNA damage signaling and repair pathways, cell cycle checkpoints, and apoptosis [1]. Highlighting the importance of the damage signaling and repair mechanisms, mutations of genes such as *ATM*, *BRCA1* and *NBS1*, involved in these mechanisms, are associated with increased DNA damage sensitivity, genomic instability, cancer predisposition, immunodeficiency, and developmental defects [2].

Mammalian Mus81 with its partners Eme1 or Eme2 form a heterodimeric structure-specific endonuclease that preferentially cleaves 3′ Flaps and replication fork intermediates [3]. This endonuclease has been shown to facilitate restart of stalled DNA replication forks by generating DNA double-strand breaks (DSBs) [4]. Mus81 also interacts with other DNA damage repair proteins including Rad54, Blm, as well as SLX4 [5]–[7]. Interestingly, Cds1, the yeast homolog of the mammalian Serine/Threonine kinase Chk2, was reported to phosphorylate and release mus81 from chromatin, presumably to prevent it from cleaving stalled replication forks (RFs) [8].

Three strains of *Mus81* mutant mice have been reported. In addition to Mus81 inactivation, *Mus81^Δex1-10^* mice have been reported to also display decreased expression of Fibulin-4 gene. Several of these homozygous mutant mice developed cardiovascular complications and died before reaching weaning age [9]. The phenotypes of these mice have been attributed to the decreased expression of Fibulin-4 [9]. *Mus81^Δex9-12^* mice have been also reported [10]. These mice displayed increased sensitivity to interstrand crosslinking (ICL) agents including MMC. Genomic instability was reported to be increased in homozygous *Mus81^Δex9-12^* MEFs expressing the human papillomavirus type 16 E6 that promotes degradation of p53. While these mutant mice were viable, they showed no increased of tumorigenesis when monitored for a period of 15 months [10]. The *Mus81^Δex3-4^* mutant mice and cells that we have generated were also highly sensitive to MMC [11]. Mice homozygous for the *Mus81^Δex3-4^* mutation showed no expression of Mus81 protein, and displayed elevated levels of spontaneous genomic instability and cancer predisposition [11]. While the cause for the lack of tumorigenesis in *Mus81^Δex9-12^* mutant mice is still not clear, inactivation of p53 in *Mus81^Δex3-4/Δex3-4^* mice rescued their MMC hypersensitivity and exacerbated their genomic instability and tumorigenesis [12].

Inactivation of MUS81 in human cells also resulted in hypersensitivity to ICL agents and elevated levels of genomic instability[13]. Importantly, MUS81 expression was found significantly decreased in human hepatocellular carcinomas, and this reduced expression correlates with a poor prognosis for patients with this cancer [14]. Moreover, a variant MUS81 allele (rs545500) was recently associated with increased risk for breast cancer [15].

CHK2 plays important roles in the DNA damage response, the signaling of the ATM-CHK2-P53 pathway and in cell cycle checkpoints including G2/M checkpoint [16], [17]. CHK2 phosphorylates a number of substrates including p53, CDC25A, CDC25C, BRCA1, E2F1, and MDC1. A role for CHK2 in cancer is supported by its rare germline or somatic mutations in certain human familial cancers and in a number of tumors and by its central role in oncogene-induced senescence [18], [19]. Interestingly, mounting evidence also supports the benefit of CHK2 inhibition in promoting tumor killing in response to genotoxic drugs [16].

Given the importance of Chk2 and Mus81 in DNA damage signaling and repair respectively, we have examined the effect of their dual inactivation on lymphoid cell differentiation, DNA damage response and cancer.

## Results/Discussion

### Chk2 Deficiency Does Not Affect Embryonic Development of *Mus81^Δex3-4/Δex3-4^* Mice

In contrast to the female specific embryonic lethality of *Mus81^Δex3-4/Δex3-4^* mice in p53 deficient background [12], *Mus81^Δex3-4/Δex3-4^Chk2^−/−^* mice were viable and born at the expected Mendelian ratio (Table S1). We next examined the fertility of *Mus81^Δex3-4/Δex3-4^Chk2^−/−^* mice and have also further assessed the viability of double mutant females. Interbreeding of *Mus81^Δex3-4/Δex3-4^Chk2^−/−^* mice as well as their breeding (males and females) to mice from different genotypes resulted in normal size litters compared to control mice (P<0.05; Table S1). In addition, interbreeding of double mutant mice or heterozygous compound mice resulted in the expected ratio of males and females (P<0.05; Table S2). Double mutant males and females were indistinguishable from their *wildtype (WT)* and single mutant littermates. Examination of the weight of 6 to 8 week old and 4 to 8 month old double mutant males and females indicated no significant differences compared to *WT* mice and single mutant controls (Figure S1A).

Collectively, these data indicate that *Mus81^Δex3-4/Δex3-4^Chk2^−/−^* males and females were viable and fertile, and their gross morphology was indistinguishable from their *WT* and single mutant littermates.

### Requirement of Chk2 and Mus81 for Homeostasis of Peripheral T and B-Cells

**Figure 1:**
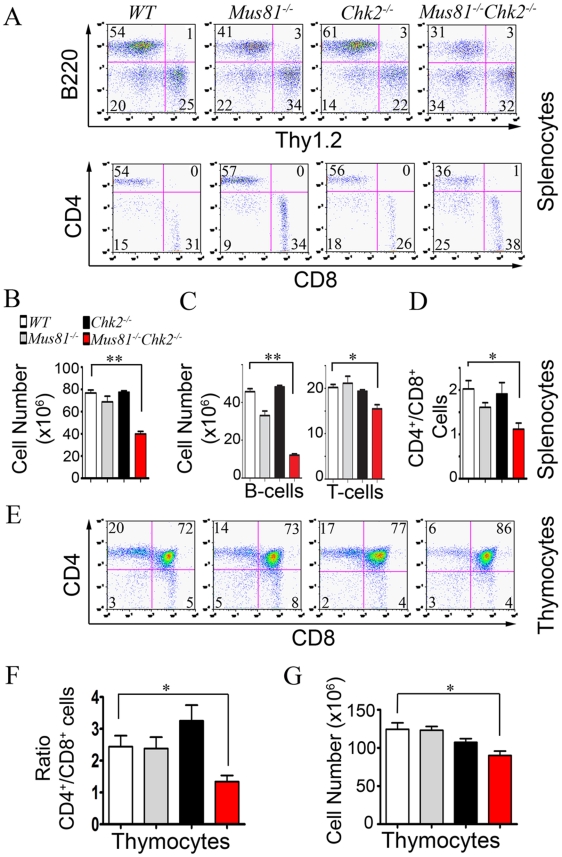
Chk2 Inactivation Impairs Differentiation and Homeostasis of *Mus81^−/−^* T and B-Cell Lineages. (A) Representative FACS analysis of total (top panel) and Thy1.2^+^ (lower panel) splenocytes from *WT*, *Mus81^Δex3-4/Δex3-4^*, *Chk2^−/−^*, and *Mus81^Δex3-4/Δex3-4^Chk2^−/−^* mice. (B and C) Number of splenocytes (B), B-cells (C, left panel) and T-cells (*C*, right panel) in spleen of *Mus81^Δex3-4/Δex3-4^Chk2^−/−^* mice and controls. (D) Ratio of CD4^+^ to CD8^+^ T-cells in spleen of *Mus81^Δex3-4/Δex3-4^Chk2^−/−^* mice and controls. (E) Representative FACS analysis of thymocytes from *Mus81^Δex3-4/Δex3-4^Chk2^−/−^* mice and controls. (F and G) Ratio of CD4^+^ to CD8^+^ thymocytes (F) and number of thymocytes (G) from each strain. At least four independent experiments using one mouse per group were performed. Percentages of the populations in A and E are indicated. Bar graphs show means ± SEM. *, *P*<0.02; **, *P*<0.0001. *Mus81^−/−^*: *Mus81^Δex3-4/Δex3-4^*.

DNA damage signaling and repair are essential for the development and function of the immune system and their failure impairs hematopoietic cell differentiation [20]. We therefore examined the effect of inactivation of Chk2 on the development and homeostasis of the lymphocytes of *Mus81^Δex3-4/Δex3-4^* mice. FACS analysis of splenocytes from 6 to 8 week old *Mus81^Δex3-4/Δex3-4^Chk2^−/−^* mice showed significant impairment of the ratio of T-cells (Thy1.2^+^) to B-cells (B220^+^) (Figure 1A and Figure S2A). The absolute number of splenocytes was significantly decreased in double mutant mice (40.9×10^6^±2.1) compared to *Mus81^Δex3-4/Δex3-4^* (68.4×10^6^±3.6; *P* = 0.0002), *Chk2^−/−^* (73.9×10^6^±3.7; *P*<0.0001) and *WT* (74.9×10^6^±2.7; *P*<0.0001) littermates (Figure 1B). The absolute number of B-cells was also significantly reduced in spleen of double mutant mice (12.4×10^6^±0.6) compared to *Mus81^Δex3-4/Δex3-4^* (32.8×10^6^±1.7; *P*<0.0001), *Chk2^−/−^* (46×10^6^±2.3; *P*<0.0001) and *WT* (44.5±1.6; *P*<0.0001) mice (Figure 1C). The absolute number of T-cells in the spleen of double mutant mice (15.9×10^6^±0.8) was also significantly decreased compared to *Mus81^Δex3-4/Δex3-4^* (21×10^6^±1.1; *P* = 0.007), *Chk2^−/−^* (19.4×10^6^±0.3; *P* = 0.02) and *WT* (19.7×10^6^±0.7; *P* = 0.01) mice (Figure 1C). Impaired T-cell lineage homeostasis in the absence of Chk2 and Mus81 was also reflected by the significantly reduced ratio of helper (CD4^+^) to cytotoxic (CD8^+^) T-cells in the spleen of double mutant mice (1.1±0.1) compared to *Mus81^Δex3-4/Δex3-4^* (1.6±0.1; *P* = 0.028), *Chk2* ^−/−^ (1.9±0.3; *P* = 0.03) and *WT* (2±0.2; *P* = 0.008) mice (Figure 1A and 1D).

To examine the impaired homeostasis of peripheral lymphocytes and the imbalance of the ratio CD4^+^ to CD8^+^ peripheral T-cells in double mutant mice, we examined the level of spontaneous and activation induced death of these cells. Spontaneous cell death was found significantly increased in both CD4^+^ and CD8^+^ naïve splenocytes from double mutant mice compared to controls (Figure S3A and S3B). In addition, LPS activation of double mutant B-cells also resulted in elevated levels of cell death compared to B-cells from single mutants or *WT* mice (Figure S7C). While the number of T-cells and B-cells were reduced in spleen of double mutant mice, the number of macrophages in these spleens remained similar to single mutants and *WT* controls.

Taken together these data demonstrate a specific role for Chk2 in maintaining homeostasis of peripheral T- and B-cells deficient for Mus81.

### Chk2 Loss Affects the Development of *Mus81^Δex3-4/Δex3-4^* Thymocytes

We next assessed the effect of dual inactivation of Chk2 and Mus81 on thymocyte development. Similar to peripheral T-cells, FACS analysis indicated a significantly decreased ratio of CD4^+^ to CD8^+^ thymocytes in *Mus81^Δex3-4/Δex3-4^Chk2* ^−/−^ mice compared to controls (Figure 1E and 1F). Moreover, total number of thymocytes was significantly reduced in double mutant mice (86.3×10^6^±5.5) compared to *Mus81^Δex3-4/Δex3-4^* (121.1×10^6^±4.1; *P* = 0.002), *Chk2* ^−/−^ (109.3×10^6^±3.9; *P* = 0.015) and *WT* (130.9×10^6^±8.7; *P* = 0.005) mice (Figure 1G).

Examination of double negative (CD4^−^CD8^−^) thymocytes and their subpopulations **DNI (CD44^+^CD25^−^)**, DNII **(CD44^+^CD25^+^)**, **DNIII (CD44^−^CD25^+^)** and DNIV **(CD44^−^CD25^−^)** from double mutant mice, single mutants and controls indicated no significant differences in cell numbers (*P*>0.05; Figure S2C). However, the numbers of CD4^+^CD8^+^ and CD4^+^ thymocytes from *Mus81^Δex3-4/Δex3-4^Chk2* ^−/−^ mice were significantly reduced compared to control littermates (Figure S2D and S2E).

We next examined the level of expression of TCRβ expression in thymocytes from the four genotypes using FACS analysis and anti-pan TCRβ chain constant region, anti-TCRVβ4, anti-TCRVβ5 and anti-TCRVβ17a. Consistent with the normal number of CD4^−^CD8^−^ thymocyte population in *Mus81^Δex3-4/Δex3-4^Chk2* ^−/−^ mice, double mutant thymocytes displayed no significant difference in the level of TCRβ expression or in the percentages of thymocytes expressing these TCRVβ compared to controls (Figure S4A and S4B). Consistent with these data, no difference was observed in TCRβ expression between *Mus81^Δex3-4/Δex3-4^Chk2* ^−/−^ and control splenocytes (Figure S4C).

These data indicate no significant changes in TCRVβ repertoire in *Mus81^Δex3-4/Δex3-4^Chk2* ^−/−^ mice compared to controls. Therefore, we conclude that inactivation of Chk2 and Mus81 did not affect TCR V(D)J recombination. Our data also indicate that while double negative thymocytes were not affected in *Mus81^Δex3-4/Δex3-4^Chk2* ^−/−^ mice, the numbers of double positive and CD4^+^ thymocytes were significantly reduced. Therefore, dual inactivation of Chk2 and Mus81 impairs thymocyte development post DN stage and this defect likely contributes to the homeostatic imbalance of peripheral T-cells in *Mus81^Δex3-4/Δex3-4^Chk2* ^−/−^ mice.

### Chk2 Is Required to Maintain the Pool of B-Cell Precursors in *Mus81^Δex3-4/Δex3-4^* Mice

**Figure 2:**
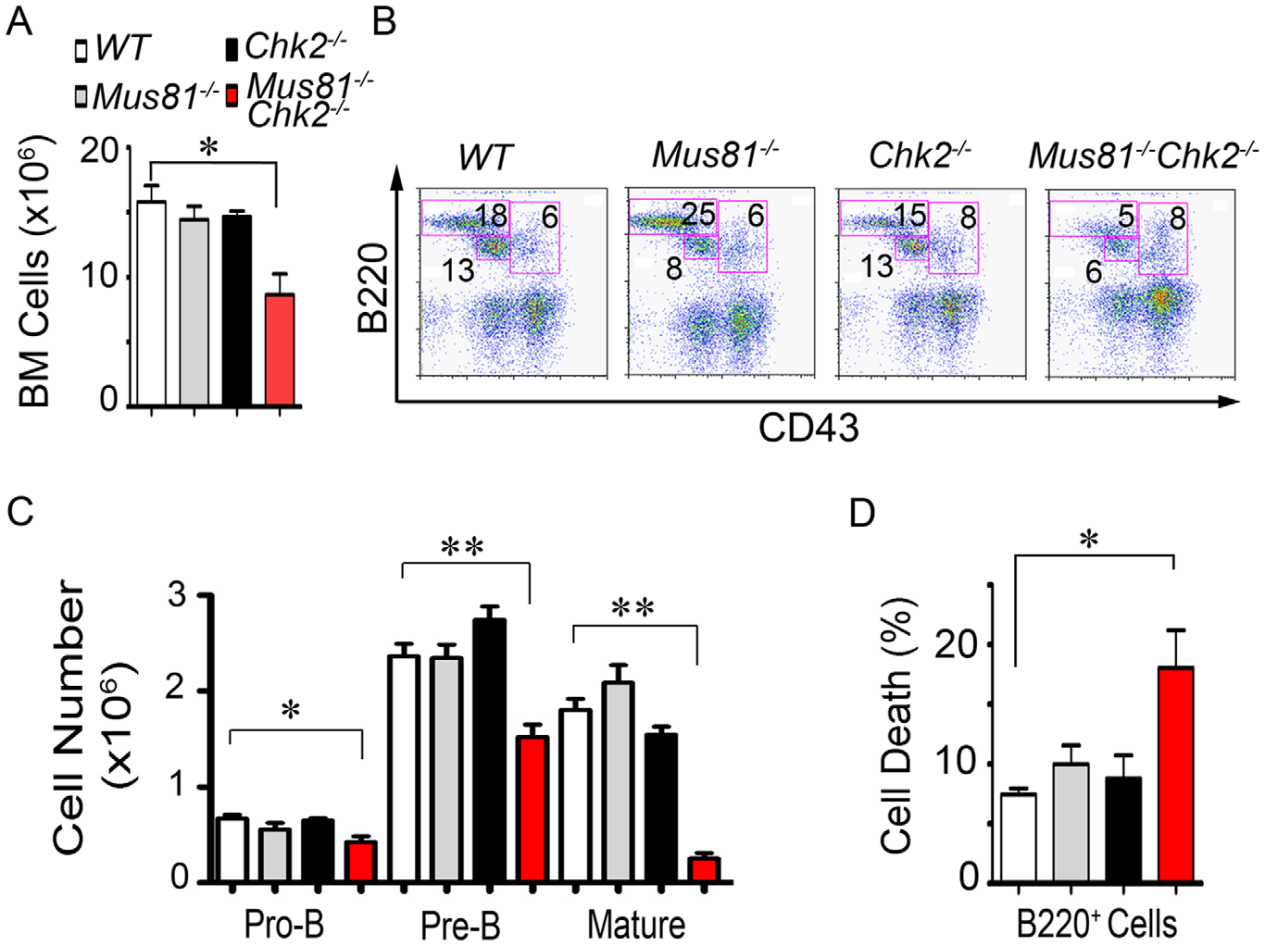
Impaired Differentiation and Increased Death of *Mus81^−/−^* B-Cells in the Absence of Chk2. (A and B) Number (A) and representative FACS analysis (B) of BM cells from *WT*, *Mus81^Δex3-4/Δex3-4^*, *Chk2^−/−^*, and *Mus81^Δex3-4/Δex3-4^Chk2^−/−^* mice. Percentages of B220^+^CD43^+^, B220^+^CD43^−^ and B220^high^CD43^−^ cells are indicated. (C and D) Number of pro-B (B220^+^CD43^+^IgM^−^), pre-B (B220^+^CD43^−^IgM^−^) and B220^high^CD43^−^IgM^+^ BM cells (C) and level of spontaneous cell death of the B220^+^ BM cells (D) from *Mus81^Δex3-4/Δex3-4^Chk2^−/−^* mice and controls. Cell death was assessed using PI staining. At least four independent experiments using one mouse per group were performed. Bar graphs show means ± SEM. *, *P*<0.05; **, *P*<0.005. *Mus81^−/−^*: *Mus81^Δex3-4/Δex3-4^*.

To determine whether the observed reduced number of B-cells in spleen of *Mus81^Δex3-4/Δex3-4^Chk2* ^−/−^ mice could result from their impaired differentiation, we examined bone marrow (BM) cell populations from double mutants and their littermate controls. Total number of BM cells was significantly reduced in double mutant mice (8.7×10^6^±1.6) compared to *Mus81^Δex3-4/Δex3-4^* (14.4×10^6^±1; *P* = 0.012), *Chk2^−/−^* (14.1×10^6^±0.7; *P* = 0.005) and *WT* (15.8×10^6^±1.3; *P* = 0.012) littermates (Figure 2A). Consistent with the decreased number of peripheral B-cells in *Mus81^Δex3-4/Δex3-4^Chk2* ^−/−^ mice, FACS analysis using antibodies against CD43, B220, IgM and IgD indicated a significantly reduced representation of mature recirculating (B220^high^CD43^−^IgM^+^, IgD^+^) B-cells in the BM of double mutant mice compared to controls (*P*<0.001; Figure 2B and 2C and Figure S5). Similarly, a significantly reduced representation of the pro-B (CD43^+^B220^+^IgM^−^) and pre-B (CD43^−^B220^+^IgM^−^) cells was also observed in *Mus81^Δex3-4/Δex3-4^Chk2* ^−/−^ mice (Figure 2B and Figure S5). Examination of absolute numbers of the pro-B, pre-B and mature B-cell populations indicated their significant decrease in double mutant mice (pro-B, *P* = 0.014; pre-B, *P* = 0.003; mature B-cells, *P* = 0.0001) compared to control littermates (Figure 2C).

To further evaluate the cause for the depletion of BM cells in the absence of Mus81 and Chk2 we examined the cell death level of B220^+^ BM cells from double mutant mice and control littermates using Propidium Iodide (PI) staining and FACS analysis. Remarkably, the level of spontaneous cell death of B220^+^ BM cells was significantly higher in *Mus81^Δex3-4/Δex3-4^Chk2* ^−/−^ mice compared to single mutants (*P*<0.05), and *WT* (*P*<0.01) controls (Figure 2D).

These data indicate that in contrast to the inactivation of either Mus81 or Chk2, their dual inactivation significantly reduces the pool of B-cell precursors. Our data also identify increased cell death as likely to contribute to the defective differentiation of B-cell lineage in *Mus81^Δex3-4/Δex3-4^Chk2* ^−/−^ mice. The earlier differentiation defect of the B-cell lineage compared to T-cell lineage of *Mus81^Δex3-4/Δex3-4^Chk2* ^−/−^ mice is likely to contribute to the more pronounced imbalance of the ratio B- to T-cells in the periphery of these mice.

### Defects in the T and B-Cell Lineages of *Mus81^Δex3-4/Δex3-4^Chk2* ^−/−^ Mice Are Cell Autonomous

In order to address whether the phenotypes observed in *Mus81^Δex3-4/Δex3-4^Chk2* ^−/−^ mice were cell autonomous or nonautonomous, we have transplanted BM cells from the 4 genotypes into *Rag1^−/−^* mice, and have examined the reconstituted mice for the number and differentiation status of BM cells, thymocytes and splenocytes (Figure S6). *Rag1^−/−^* mice arrest their B-cell development at the Pro-B stage [21]. Examination of the number of BM cells in the *Rag1^−/−^* mice reconstituted with *Mus81^Δex3-4/Δex3-4^Chk2* ^−/−^ BM cells indicated no significant differences at the Pre-B-stage (Figure S6A). However, similar to *Mus81^Δex3-4/Δex3-4^Chk2* ^−/−^ mice, reconstituted *Rag1^−/−^* mice with *Mus81^Δex3-4/Δex3-4^Chk2* ^−/−^ BM cells also displayed significantly reduced number of recirculating B-cells compared to reconstituted controls (*P*<0.006; Figure S6B).

*Rag1^−/−^* mice reconstituted with *Mus81^Δex3-4/Δex3-4^Chk2* ^−/−^ BM cells, similar to double mutant mice, displayed reduced number of thymocytes (*P*<0.03; Figure S6C). In addition, spleen from these mice also displayed reduced number of total splenocytes, B-cells, T-cells, CD4^+^ T-cells and CD8^+^ T-cells (*P*<0.05; Figure S6D).

These data indicate that the phenotypes observed with BM, thymus and spleen of *Mus81^Δex3-4/Δex3-4^Chk2* ^−/−^ mice were largely cell autonomous.

### Chk2 Inactivation Does Not Rescue MMC Hypersensitivity of *Mus81^Δex3-4/Δex3-4^* T-Cells

**Figure 3:**
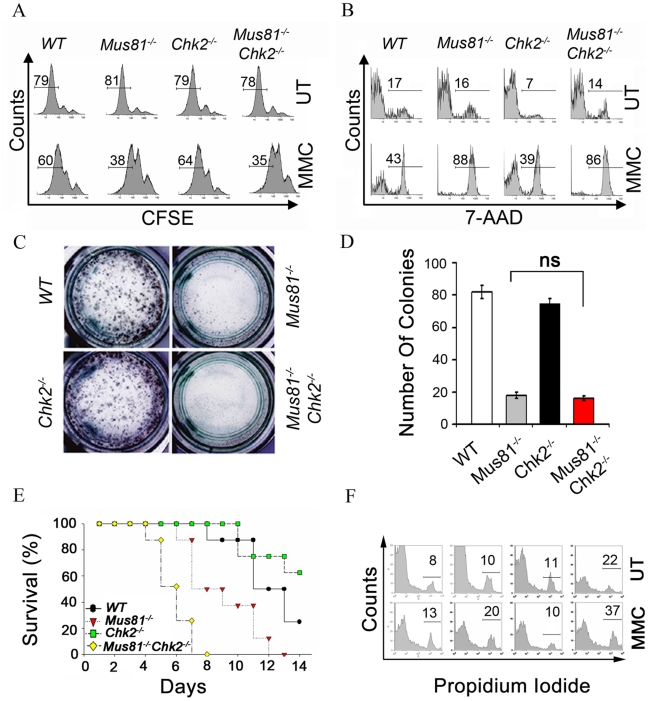
Effect of Chk2 Inactivation on MMC Sensitivity of *Mus81^−/−^* Cells and Mice. (A) Representative CFSE staining of *WT*, *Mus81^Δex3-4/Δex3-4^*, *Chk2^−/−^*, and *Mus81^Δex3-4/Δex3-4^Chk2^−/−^* activated T-cells (anti-CD3 + IL2) 120hr post CFSE labelling. Cells were either untreated (UT) or treated with MMC (0.1 µg/ml) for the last 72 hr (MMC). Percentage of cells achieving two cell divisions is shown. (B) Representative FACS analysis showing the level of cell death (7AAD^+^) of activated T-cells either untreated (UT) or 48 hr post MMC (0.5 µg/ml) treatment. Percent of dead cells is shown. (C and D) BM colony forming assay. BM cells were plated on media containing 0 or 40 ng/ml of MMC and colonies were counted 10 days post-treatment. Representative picture of colonies at day 10 of MMC treatment is shown (C). Data normalized to untreated cells is presented as the mean ± SD of three independent experiments (D). No statistical significance: ns. (E) Kaplan-Meier survival curve of *WT*, *Mus81^Δex3-4/Δex3-4^*, *Chk2^−/−^*, and *Mus81^Δex3-4/Δex3-4^Chk2^−/−^* cohort of mice (n = 10 each) in response to i.p injection of MMC (12.5 mg/kg). (F) Representative FACS analysis of cell death using PI staining and B220 gated BM cells from *Mus81^Δex3-4/Δex3-4^Chk2^−/−^* mice and controls. Mice were either untreated (UT) or received MMC (12.5 mg/kg) injection three days prior to analysis (MMC). Data shown is representative of at least three independent experiments. *Mus81^−/−^*: *Mus81^Δex3-4/Δex3-4^*.

Hypersensitivity to ICL agents including MMC was reported for *Mus81^Δex1-10^* [4], *Mus81^Δex9-12^* [10] as well as with *Mus81^Δex3-4^* mutations [11]. The hypersensitivity of *Mus81^Δex3-4/Δex3-4^* cells was rescued in p53 null background [12]. Examination of the proliferation of activated T-cells (anti-CD3 + IL2) using the Carboxyl fluoroscein succinimidyl ester (CFSE) assay indicated similar level of cell divisions in T-cells from *Mus81^Δex3-4/Δex3-4^Chk2* ^−/−^ mice and control mice (Figure 3A). However, 72 hours (hr) post-MMC treatment (0.1 µg/ml), double mutant cells exhibited a pronounced proliferative defect that was similar to the level observed for *Mus81^Δex3-4/Δex3-4^* cells (Figure 3A). Moreover, loss of Chk2 also failed to rescue MMC induced G2 arrest and apoptosis of *Mus81^Δex3-4/Δex3-4^* T-cells (Figure 3B and Figure S7A).

We also examined the effect of Mus81 inactivation on the radioresistance of *Chk2^−/−^* activated T-cells. A nearly equivalent resistance was observed in both *Mus81^Δex3-4/Δex3-4^Chk2* ^−/−^ and *Chk2^−/−^* cells 12 hr post ionizing radiation (IR; 4Gy) (Figure S7B), supporting that Mus81 inactivation has no effect on the radioresistant phenotype of *Chk2^−/−^* cells.

Therefore, in contrast to p53 inactivation [12], inactivation of Chk2 failed to rescue MMC hypersensitivity of *Mus81^Δex3-4/Δex3-4^* T-cells.

### Effect of Chk2 Inactivation on MMC Hypersensitivity of *Mus81^Δex3-4/Δex3-4^* BM Cells and Mice

To examine whether Chk2 plays a role in the MMC hypersensitivity of *Mus81^Δex3-4/Δex3-4^* BM cells [12], we performed colony forming assay, in the absence or presence of MMC (40 ng/ml), using double mutant, *Mus81^Δex3-4/Δex3-4^*, *Chk2^−/−^* and *WT* BM cells. Twelve days post plating, MMC treated BM cells from *Chk2^−/−^* and *WT* mice formed a similar number of colonies (Figure 3C and 3D). However, similarly to *Mus81^Δex3-4/Δex3-4^* BM cells, double mutant BM cells displayed reduced colony-forming capacity post-MMC treatment (Figure 3C and 3D). Therefore, inactivation of Chk2 in *Mus81^Δex3-4/Δex3-4^* BM cells does not rescue their MMC hypersensitivity.

Given the high *in vivo* MMC sensitivity of *Mus81^Δex3-4/Δex3-4^* mice [11] and *Mus81^Δex9-12^* mice [10], we assessed the effect of Chk2 inactivation on the MMC hypersensitivity of *Mus81* mutant mice. Cohorts of mice from the four genotypes received intraperitoneal (i.p) injection of MMC (12.5 mg/kg) and were monitored for survival (Figure 3E). In contrast to the results of the *in vitro* BM colony forming assay suggestive of equivalent MMC sensitivity of double mutant and *Mus81^Δex3-4/Δex3-4^* BM cells, double mutant mice displayed significantly higher sensitivity to MMC (mean survival  = 6 days) than *Mus81^Δex3-4/Δex3-4^* mice (mean survival  = 9 days).

As BM failure might contribute to the elevated MMC sensitivity of *Mus81^Δex3-4/Δex3-4^Chk2^−/−^* mice, we examined the *in vivo* effects of MMC on BM cell populations from *Mus81^Δex3-4/Δex3-4^Chk2* ^−/−^ mice, single mutants and *WT* littermates. Mice were either left untreated or subjected to i.p injection of MMC (12.5 mg/kg) and the level of death of B220^+^ BM cells was examined three days later using PI staining. Increased cell death was observed in B220^+^ BM cells from both untreated and MMC treated double mutant mice compared to control littermates (Figure 3F).

These data indicate that in contrast to p53 inactivation [12], loss of Chk2 synergizes MMC hypersensitivity of *Mus81^Δex3-4/Δex3-4^* mice and BM cells.

### Increased Mitotic Catastrophe of *Mus81^Δex3-4/Δex3-4^Chk2* ^−/−^ Cells

Mitotic catastrophe is an abnormal mitosis that triggers the death of cells with damaged DNA as they enter mitosis [22]. Mitotic catastrophe is also triggered by agents that affect the stability of microtubules as well as by mitotic failure that results from impaired cell cycle checkpoints. Chk2, which is important for G2/M checkpoint, has been reported to negatively regulate mitotic catastrophe of DNA damaged cells [23]. As *Mus81^Δex3-4/Δex3-4^Chk2* ^−/−^ cells displayed increased levels of spontaneous cell death and these mutant cells and mice also displayed elevated MMC-induced cell death, we have examined whether cell death of *Mus81^Δex3-4/Δex3-4^Chk2* ^−/−^ cells is triggered by mitotic catastrophe. Cells undergoing mitotic catastrophe typically display micronuclei, increased frequency of giant cells, multilobed nuclei, and nuclear bridging [22].

**Figure 4:**
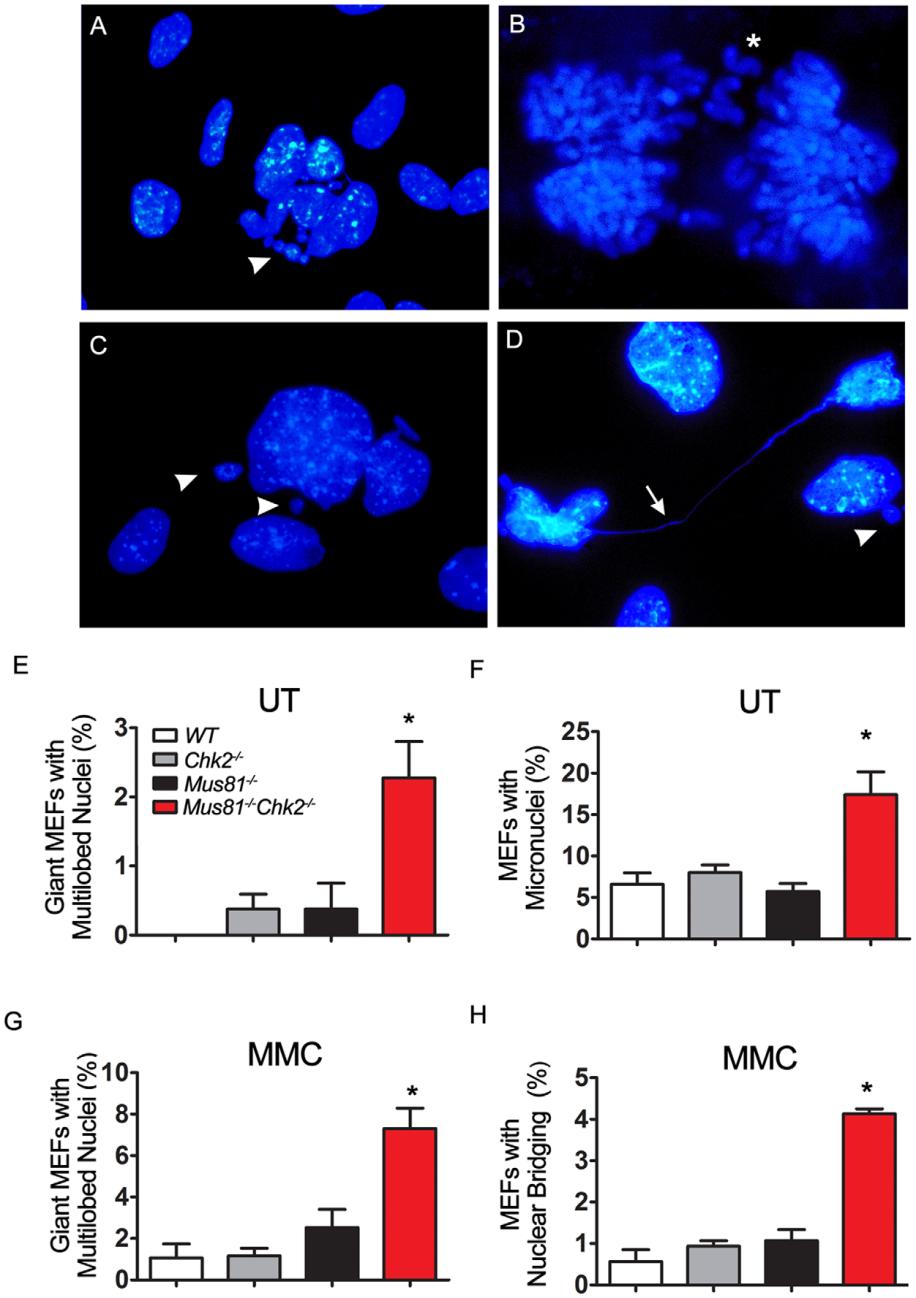
Loss of Chk2 Triggers Mitotic Catastrophe of *Mus81^−/−^* Cells. (A) Representative giant *Mus81^Δex3-4/Δex3-4^Chk2^−/−^* MEFs with multilobed nuclei (arrow head). (B) Representative abnormal metaphase with lagging chromosomes (star) in *Mus81^Δex3-4/Δex3-4^Chk2^−/−^* MEFs. (C) Representative MMC treated *Mus81^Δex3-4/Δex3-4^Chk2^−/−^* MEFs showing a giant cell with multilobed nuclei and micronuclei (arrowhead). (D) Representative MMC treated *Mus81^Δex3-4/Δex3-4^Chk2^−/−^* MEFs showing nuclear bridging (arrow). (E) Percentage of giant cells with multilobed nuclei in untreated *WT, Mus81^Δex3-4/Δex3-4^, Chk2^−/−^, Mus81^Δex3-4/Δex3-4^Chk2^−/−^* primary MEFs. (F) Percentage of untreated primary MEFs with micronuclei. (G) Percentage of giant cells with multilobed nuclei in primary MEFs post 24 hr of MMC treatment. (H) Percentage of MMC treated primary MEFs with nuclear bridging. *: *P*<0.02. UT: untreated. *Mus81^−/−^*: *Mus81^Δex3-4/Δex3-4^*.

Untreated *Mus81^Δex3-4/Δex3-4^Chk2* ^−/−^ primary MEFs stained with DAPI displayed increased frequency of abnormal giant cells with multilobed nuclei compared to controls MEFs (*P*<0.025; Figure 4A and 4E). Lagging chromosomes were also observed in untreated *Mus81^Δex3-4/Δex3-4^Chk2* ^−/−^ MEFs (Figure 4B). The frequency of untreated double mutant MEFs displaying micronuclei was also elevated compared to controls (*P*<0.018; Figure 4F). In response to MMC, *Mus81^Δex3-4/Δex3-4^Chk2* ^−/−^ MEFs displayed increased frequency of giant cells with multilobed nuclei (*P*<0.01; Figure 4G) as well as cells with nuclear bridging (*P*<0.001; Figure 4D and 4H). These data indicate increased mitotic catastrophe of *Mus81^Δex3-4/Δex3-4^Chk2* ^−/−^ cells.

Loss or reduction of the expression of survivin [24], an inhibitor of apoptosis protein (IAP), has been shown to lead to cell death by mitotic catastrophe [25]. We have therefore performed Western blot analyses to examine the levels of survivin in untreated or 18 hr and 24 hr post-MMC treatment of LPS activated B-cells from *Mus81^Δex3-4/Δex3-4^Chk2* ^−/−^ mice, single mutants and *WT* controls. We observed a lower level of survivin in MMC treated *Mus81^Δex3-4/Δex3-4^Chk2* ^−/−^ B-cells compared to single mutants and *WT* controls (Figure S8). We propose that decreased survivin level in *Mus81^Δex3-4/Δex3-4^Chk2* ^−/−^ cells could contribute to their mitotic catastrophe.

Increased mitotic catastrophe would trigger cell death of *Mus81^Δex3-4/Δex3-4^Chk2* ^−/−^ cells and therefore is likely to contribute to the impaired homeostasis of BM cells, thymocytes and splenocytes in double mutant mice.

### Impaired of Activation of p53 and Defective G2/M Checkpoint in *Mus81^Δex3-4/Δex3-4^Chk2* ^−/−^ Cells

**Figure 5:**
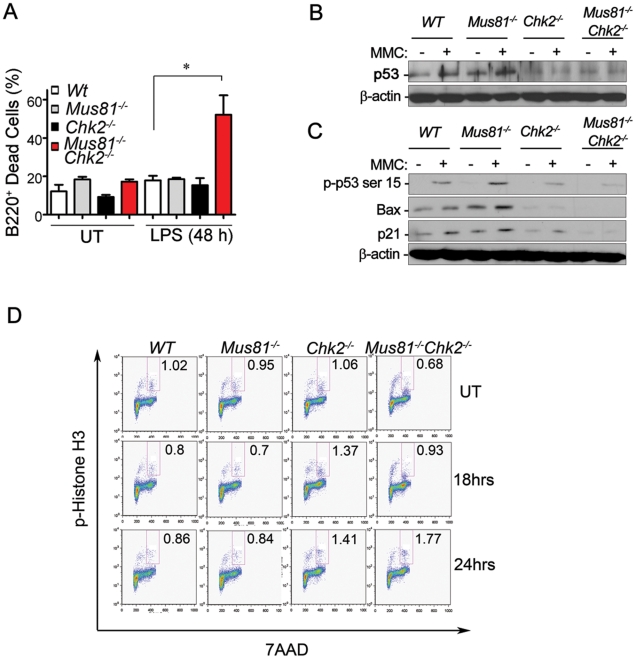
Loss of Chk2 Impairs p53 Activation and G2/M Checkpoint in *Mus81^−/−^* Cells. (A) The cell death level of activated *Mus81^Δex3-4/Δex3-4^Chk2^−/−^* B-cells and controls. Data is presented as the mean ± SD of four independent experiments. * *P* = 0.016. (B) Representative Western blot analysis of total p53 in LPS activated B-cells untreated (−) or treated with MMC (+). (C) Representative Western blot analysis performed as indicated in (B) shows the expression level of Serine 15-p53 (p-p53 ser 15), Bax, p21 and β-actin. (D) Representative FACS analysis of LPS activated B-cells either untreated or treated for 18 hr or 24 hr with MMC. Percentage of mitotic cells (p-Histone H3^+^) is indicated. *Mus81^−/−^*: *Mus81^Δex3-4/Δex3-4^*.

p53 plays a central role in DNA damage responses and its phosphorylation by Chk2 on Serine 20 is important for its stability and activation [26]. Therefore, we examined the level of p53 expression and activation in double mutants and control B-cells from the four genotypes. While LPS activated double mutant B-cells displayed increased cell death levels (Figure 5A), the basal level of p53 in these activated B-cells remained similarly low compared to single mutants and *WT* B-cells (Figure 5B). MMC treatment of *WT* and *Mus81^Δex3-4/Δex3-4^* activated B-cells increased p53 expression levels (Figure 5B). However, double mutant and *Chk2* ^−/−^ cells failed to increase their level of p53 in response to MMC treatment (Figure 5B). In accordance with the low level of p53 in untreated and MMC treated double mutant cells, the level of Serine 15-p53 (a substrate for ATM), and the levels of Bax and p21 (p53 downstream targets) were low to undetectable in double mutant cells under untreated conditions and were not induced in response to MMC (Figure 5C). In contrast, the expression levels of Serine 15-p53, Bax and p21 in *Mus81^Δex3-4/Δex3-4^* and *WT* cells were significantly increased in response to MMC (Figure 5C). These data indicate that despite the lack of p53 activation in double mutant B-cells, these cells displayed elevated level of spontaneous and MMC-induced death, supporting that this death is p53- independent. Consistent with these data, mitotic catastrophe has been shown to take place in a p53-independent manner [27].

Chk2 is required for the enforcement of the G2/M cell cycle checkpoint following DNA damage [16], [18]. It has been shown that loss of Chk2 expression or inhibition of its kinase activity sensitizes cells to death during mitosis [23]. Therefore, we have examined G2/M cell cycle checkpoint in untreated and MMC-treated double mutant and control activated B-cells (Figure 5D). While the fraction of mitotic cells (positive for phospho-Histone H3) was reduced 18 hr and 24 hr post MMC treatment in *WT* and *Mus81^Δex3-4/Δex3-4^* B-cells, both *Chk2^−/−^* and *Mus81^Δex3-4/Δex3-4^Chk2* ^−/−^ B-cells failed to activate G2/M checkpoint in response to MMC treatment as indicated by their increased fraction of phospho-Histone H3 positive cells compared to untreated controls (Figure 5D).

These data indicate that despite MMC-induced DNA damage, double mutant cells are allowed to enter mitosis, due to their impaired G2/M checkpoint, therefore resulting in mitotic catastrophe and cell death.

### Effect of Chk2 Inactivation on Genomic Instability of *Mus81^Δex3-4/Δex3-4^* Cells

*Mus81^Δex3-4/Δex3-4^* mice display elevated levels of genomic instability [11], likely due to their defective repair of stalled RFs [28]. Genomic instability was also increased in *Mus81^Δex9-12/Δex9-12^* MEFs expressing the oncoprotein E6 [10] and in *Mus81^Δex1-10/Δex1-10^* ES cells treated with Hydoxyurea [28]. In addition, inactivation of MUS81 by gene targeting in the human colon cancer cell line HCT116 also increased the level of genomic instability of these cells [13].

**Figure 6:**
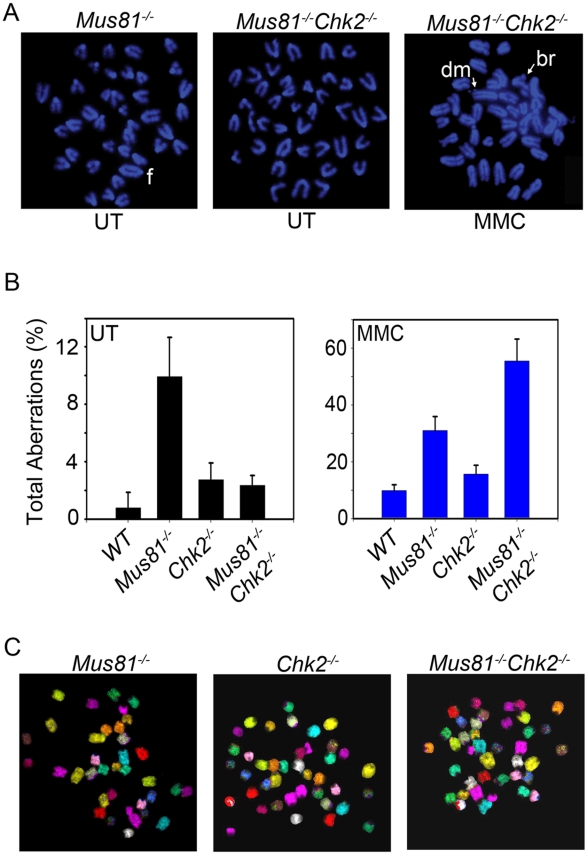
Effects of Chk2 Inactivation on Genomic Instability of *Mus81^−/−^* Cells. (A) Representative metaphases of LPS activated *Mus81^Δex3-4/Δex3-4^* and *Mus81^Δex3-4/Δex3-4^Chk2^−/−^* B-cells either untreated (UT) or post-MMC treatment (MMC). f: chromosomal fusion, dm: double minute and br: break. (B) Incidence of total spontaneous (UT, left panel) and MMC-induced chromosomal aberrations (MMC, right panel). Data is presented as the mean ± SD of three independent experiments. A minimum of 50 metaphase spreads were analyzed for each genotype, treatment and experiment. (C) mFISH performed on metaphase spreads from *Mus81^Δex3-4/Δex3-4^, Chk2^−/−^* and *Mus81^Δex3-4/Δex3-4^Chk2^−/−^* cells. *Mus81^−/−^*: *Mus81^Δex3-4/Δex3-4^*.

While Chk2 inactivation resulted in increased genomic instability of *Brca1^−/−^* cells, it did not for *Nbs1^−/−^* or *Mre11^−/−^* cells [29], [30]. To determine the effect of Chk2 inactivation on the increased spontaneous genomic instability associated with *Mus81^Δex3-4/Δex3-4^* mutation [11], we examined metaphase spreads of activated B-cells from the different genotypes. These analyses indicated elevated level of spontaneous genomic instability of *Mus81^Δex3-4/Δex3-4^* B-cells while *Chk2^−/−^* B-cells exhibited only a slightly increased level of spontaneous genomic instability (Figure 6A and 6B; Table S3). Remarkably, while inactivation of p53 exacerbated spontaneous genomic instability of *Mus81^Δex3-4/Δex3-4^* cells [12], inactivation of Chk2 in these cells significantly suppressed their spontaneous genomic instability. Multicolor fluorescence in situ hybridization (mFISH) also failed to detect any interchromosomal aberrations like translocations in *Mus81^Δex3-4/Δex3-4^Chk2* ^−/−^ cells and their controls (Figure 6C). These data suggest that either Chk2 inactivation decreases the survival of *Mus81^−/−^* cells that carry genomic instability, or alternatively that it prevents genomic instability from occurring in *Mus81* mutants. However, the latter is inconsistent with the drastic increased genomic instability of MMC-treated double mutant B-cells compared to *Mus81^Δex3-4/Δex3-4^* controls (Figure 6A and 6B; Table S3).

To examine whether the reduced level of spontaneous genomic instability of *Mus81^Δex3-4/Δex3-4^* B-cells in Chk2 null background was restricted to these cells or could be observed in other cell types, we have examined metaphase spreads from activated T-cells and primary MEFs from the four genotypes. Similar to B-cells, *Mus81^Δex3-4/Δex3-4^Chk2* ^−/−^ T-cells (Table S4) and MEFs (Table S5) displayed reduced levels of spontaneous genomic instability compared to *Mus81^Δex3-4/Δex3-4^* controls. In addition, in response to MMC treatment, similar to double mutant-B-cells, *Mus81^Δex3-4/Δex3-4^Chk2* ^−/−^ T-cells exhibited elevated levels of genomic instability compared to their *Mus81^Δex3-4/Δex3-4^* controls (Table S4).

The increased genomic instability of MMC treated *Mus81^Δex3-4/Δex3-4^Chk2* ^−/−^ cells compared to *Mus81^Δex3-4/Δex3-4^* cells is consistent with their failure to activate G2/M checkpoint. Despite their DNA damage, these double mutant cells enter mitosis where they suffer mitotic catastrophe. Under normal conditions, mitotic catastrophe could serve to trigger cell death and eliminate *Mus81^Δex3-4/Δex3-4^Chk2* ^−/−^ cells carrying damaged DNA. Therefore, increased mitotic catastrophe of *Mus81^Δex3-4/Δex3-4^Chk2* ^−/−^ cells could allow to suppress spontaneous genomic instability. Under chronic conditions of DNA damage (presence of MMC), *Mus81^Δex3-4/Δex3-4^Chk2* ^−/−^ cells accumulate excessive DNA damage due to their impaired G2/M checkpoint (Chk2 inactivation) and defective DNA damage repair (Mus81 inactivation), thus leading to accumulation of chromosomal aberrations. However, these cells are ultimately eliminated as indicated by the increased MMC sensitivity of *Mus81^Δex3-4/Δex3-4^Chk2* ^−/−^ mice (Figure 3E), increased mitotic catastrophe (Figure 4) and cell death (Figure 3F) of MMC double mutant treated cells.

### Inactivation of Chk2 Suppresses Tumorigenesis of *Mus81^Δex3-4/Δex3-4^* Mutants

CHK2 is mutated in certain human familial cancers and in a number of other tumors [18]. Tumorigenesis was also observed in a mouse model for *CHK2 del1100C*, a mutation associated with increased cancer risk in humans [31]. In addition, inactivation of Chk2 increased cancer risk of *Brca1*, *Nbs1* and *Mre11* mutant mice [30]. Stracker et al. demonstrated that inactivation of Chk2 in DNA-PKcs deficient background did not predispose these mice for cancer, and proposed that Chk2 suppresses the oncogenic potential of DNA damage arising in the S and G2 but not G1 phases of the cell cycle [30].

**Figure 7:**
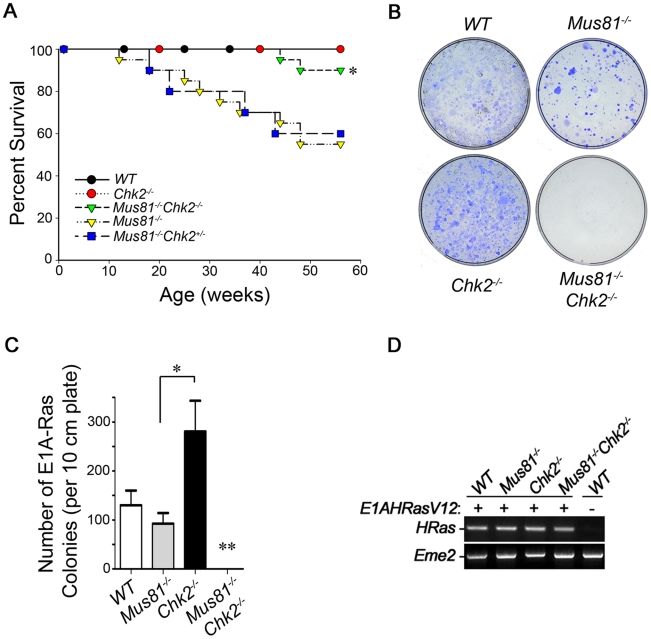
Inactivation of Chk2 Suppresses Tumorigenesis and Oncogenic Transformation of *Mus81^−/−^* Mutants. (A) Kaplan-Meier analysis percent of the survival for *WT* (n = 20), *Mus81^Δex3-4/Δex3-4^* (n = 20), *Chk2^−/−^* (n = 20), *Mus81^Δex3-4/Δex3-4^Chk2^+/−^* (n = 10) and *Mus81^Δex3-4/Δex3-4^Chk2^−/−^* (n = 20) cohort mice monitored for 1 year. Log Rank test statistical analysis of survival curves; * *P* = 0.02. (B and C) *WT*, *Mus81^Δex3-4/Δex3-4^*, *Chk2^−/−^*, and *Mus81^Δex3-4/Δex3-4^Chk2^−/−^* primary MEFs were infected with a retrovirus coexpressing E1A and Ras and selected with puromycin. Three weeks post-infection colonies were counted. (B) Representative pictures of colonies at day 21 post-infection and the number of colonies counted are shown (C). Data are representative of three independent experiments. * *P<0.05*. ** No E1A-Ras transformed *Mus81^Δex3-4/Δex3-4^Chk2^−/−^* colonies were observed in three independent experiments. (D) Representative PCR analysis confirming integration of *E1A*-*HRasV12* in MEFs infected with *E1A*-*HRasV12I* retrovirus for 10 days. HRas specific primers were used. Eme2 primers were used for PCR control. *Mus81^−/−^*: *Mus81^Δex3-4/Δex3-4^*.

*Mus81^Δex3-4/Δex3-4^* mice have increased risk for cancer [11], and this cancer risk is further exacerbated in *p53^−/−^* background [12]. In order to address the effect of Chk2 inactivation on tumorigenesis of *Mus81* mutant mice, cohorts of *Mus81^Δex3-4/Δex3-4^Chk2^−/−^*, *Mus81^Δex3-4/Δex3-4^Chk2^+/−^*, *Mus81^Δex3-4/Δex3-4^*, *Chk2^−/−^* and *WT* mice were monitored for survival and tumorigenesis for a period of one year (Figure 7A). As expected, tumors were not observed in *WT* and *Chk2^−/−^* mice in accordance with our previous studies [11], , *Mus81^Δex3-4/Δex3-4^* mice displayed increased risk for tumors and only 55% (11/20) of the monitored *Mus81^Δex3-4/Δex3-4^* mice were viable and tumor-free at the end of one year. Remarkably, loss of Chk2 dramatically rescued tumor susceptibility of *Mus81^Δex3-4/Δex3-4^* mice as 90% of double mutant mice (18/20) were viable and tumor-free at the end of one year (*P* = 0.02*;* double mutants versus *Mus81^Δex3-4/Δex3-4^* mice). The two double mutant mice that did not survive the one year observation period died of infection. *Mus81^−/−^* mice in Chk2 null background were however not fully protected from tumorigenesis as three double mutant mice (6%) developed tumors past 15 months of age.

Histological analyses demonstrated that tumors developed in *Mus81^Δex3-4/Δex3-4^* mice (1 year old or younger) were B-cell lymphoma (84%), large T-cell lymphoma (8%) and osteocarcinoma (8%). Histological examination of the three tumors that developed in *Mus81^Δex3-4/Δex3-4^Chk2^−/−^* mice past 15 months of age, indicated that one of the tumors was a sarcoma while the two others were B-cell lymphomas.

The remarkable mitigation of tumorigenesis of *Mus81^Δex3-4/Δex3-4^* mice by Chk2 inactivation parallels their reduced level of spontaneous genomic instability, failure of G2/M checkpoint and increased mitotic catastrophe. Our finding is consistent with a beneficial effect of CHK2 inhibition on tumor responses to genotoxic chemotherapeutic drugs [18].

### Chk2 Inactivation Suppresses Oncogenic Transformation of *Mus81^Δex3-4/Δex3-4^* Cells

Dysregulation of oncogenes, similar to tumor suppressors, plays critical roles in human cancer. Chk2 is activated by oncogenes such as Ras, and is important for mediating oncogene-induced senescence, a safeguard against cancer development [32]. In addition, Chk2 inactivation has been shown to promote oncogenic transformation [19].

We therefore investigated the effect of Chk2 inactivation on transformation of *Mus81^Δex3-4/Δex3-4^* cells and examined oncogenic transformation of double mutant primary MEFs, single mutants and *WT* controls. Cells were infected with a retrovirus expressing E1A and Ras and transformed colonies were counted three weeks later (Figure 7B and 7C). Consistent with previous studies [19], the number of E1A-Ras transformed colonies formed by *Chk2^−/−^* MEFs was elevated compared to *WT* controls (*P<0.05*). Strikingly, while *Mus81^Δex3-4/Δex3-4^* MEFs were able to form E1A-Ras transformed colonies at a similar frequency compared to *WT* MEFs (*P* = 0.36), no E1A-Ras transformed colonies were obtained from *Mus81^Δex3-4/Δex3-4^Chk2^−/−^* MEFs in three independent experiments.

To confirm that the lack of transformation of *Mus81^Δex3-4/Δex3-4^Chk2^−/−^* MEFs is due to the loss of Mus81 and Chk2 and not due to the inability of these MEFs to be infected with pBabe *E1A*-*HRasV12* retrovirus, MEFs from the four genotypes were infected with this retrovirus and genomic DNA of these cells was prepared 7 days post puromycin selection and PCR analysis was performed to assess the presence of *E1A*-*HRasV12* in the extracted genomic DNA. These analyses demonstrated that despite integration of *E1A*-*HRasV12* in the genome of *Mus81^Δex3-4/Δex3-4^Chk2^−/−^* MEFs (Figure 7D), these cells failed to be transformed.

These data demonstrate that similar to the suppression of spontaneous tumorigenesis of *Mus81^Δex3-4/Δex3-4^* mice, Chk2 inactivation also suppresses oncogenic transformation of *Mus81^Δex3-4/Δex3-4^* cells.

Collectively our data demonstrate an important role for Chk2 in maintaining homeostasis of BM cells, thymocytes and splenocytes deficient for Mus81. Remarkably, inactivation of Chk2 also reduced spontaneous genomic instability associated with *Mus81* mutation, and significantly protected *Mus81* mutants from tumorigenesis and oncogenic transformation. We also report increased mitotic catastrophe in double mutant cells, likely due to the inactivation of G2/M checkpoint associated with loss of Chk2 and increased spontaneous DNA damage associated with loss of Mus81. The phenotypes observed in *Mus81^Δex3-4/Δex3-4^Chk2^−/−^* mice are p53-independent. In contrast to *Mus81^Δex3-4/Δex3-4^Chk2^−/−^* cells, *Mus81^Δex3-4/Δex3-4^p53^−/−^* cells retain proficient G2/M checkpoint that prevents their entry to mitosis in the presence of damaged DNA. Therefore, we propose that G2/M checkpoint failure and increased mitotic catastrophe are the mechanisms that result in reduced spontaneous genomic instability, tumorigenesis and oncogenic transformation.

Our data provide *in vivo* evidence that inhibition of Chk2 can have remarkable inhibitory effects on tumorigenesis. While pharmacological inhibitors of CHK2 are being considered for cancer therapy [18], our data suggest that the therapeutic effect of such inhibitors is likely to depend on the genetic background of the tumors. Namely, while Chk2 inactivation promotes tumorigenesis of *Brca1*, *Nbs1* and *Mre11* mutant strains, it remarkably suppresses cancer in *Mus81^Δex3-4/Δex3-4^* mutant background. Our preclinical data highly support the potential therapeutic value for CHK2 or MUS81 inhibitors for cancer patients with *MUS81* or *CHK2* mutations, respectively.

## Materials and Methods

### Mice

*Mus81^Δex3-4/Δex3-4^Chk2^−/−^* mice were obtained by crossing *Mus81* [11] and *Chk2* [33] mutant mice. Mice were in a mixed 129/J × C57BL/6 genetic background and were genotyped by PCR (conditions available upon request).

### Flow Cytometry

BM, thymus and spleen cells from 6–8 week-old mice were stained with antibodies (Pharmingen) against B220, CD43, IgM, IgD, Thy1.2, TCR, CD4, CD8, CD44, CD25 and TCRVβ (4, 5.1–5.2, and 17a). Fluorescence-activated cell sorting (FACS) analyses were performed using a FACS Calibur (Becton Dickinson).

### Cell Cycle Analysis

Peripheral T-cells stimulated with anti-CD3 and IL-2 (50 U/mL) for 48 h, were treated with 0.5 µg/ml MMC (Sigma) for 18 hr. Cells were washed three times with PBS and cultured for an additional 18 hr. Cells were fixed in 70% ethanol, and DNA was stained with 5 µg/mL of PI (Sigma). Cells at the G1, S, and G2/M phases of the cell cycle were determined using FLOWJO analysis software.

### Phospho-Histone H3 Staining

Peripheral B-cells activated with LPS (10 µg/ml) for 48 hr, were treated with 0.1 µg/ml of MMC (Calbiochem) for 18 hr. Cells fixed and permeabilized with ice cold methanol were stained with anti-phospho Histone-H3 ser10 FITC (Cell Signaling). Cells were analysed using FACS Calibur (Becton Dickinson) and results were analysed using FLOWJO analysis software.

### CFSE Staining

Peripheral T-cells were stained with 5 µM CFSE and then activated for two days with plate bound anti-CD3 in the presence of IL-2. Untreated and 0.1 µg/ml MMC treated cells were grown for an additional 72 hr and analyzed by flow cytometry.

### MEFs and Retrovirus Infection

Primary MEFs (3×10^5^) were cultured in DMEM plus 10% FCS in the presence of pBabe *E1A*-*HRasV12* retrovirus and 8 µg/ml of polybrene. At day three post-infection, Puromycin selection (2 µg/ml) was carried out for three weeks. Colonies on the plates were fixed with ice cold methanol, stained with 0.5% crystal violet and counted. To confirm that MEFs have integrated *E1A*-*HRasV12,* genomic DNA was prepared from infected cells post 7 days of puromycin selection. PCR was then performed on MEFs genomic DNA using specific primers for *HRasV12* (F: CGGAATATAAGCTGGTGGTG and R: CGGTATCCAGGATGTCCAAC). Eme2 primers (F: ACGGCTTCCCTACCAGCACA and R: AGTGGCTGCTACTCGGCTTCA) were used as controls for the PCR.

### Chromosomal Aberrations Analysis

LPS (10 µg/ml) activated splenocytes were cultured for 48 hr in the presence or absence of MMC (40 ng/ml) and metaphase spreads prepared as previously described [11]. Chromosomal aberrations were determined for a minimum of 60 metaphase spreads per cell type. In mFISH, all 21 chromosomes are each painted in a different color using combinatorial labeling and mFISH probe kit (MetaSystems) as previously described [34].

T-cells were activated with anti-CD3 (10 µg/mL) + IL2 (50 U/mL) for 48 hr in the presence or the absence of MMC (40 ng/ml). Metaphase spreads were prepared as described [11]. Chromosomal aberrations were determined for a minimum of 100 metaphase spreads.

Metaphase spreads of primary MEFs were similarly prepared and chromosomal aberrations were determined for a minimum of 50 metaphase spreads.

Percent aberrations are calculated as aberrations per metaphase then percentages are determined. Breaks/Fragments include chromosome breaks, chromatid breaks, and chromosome fragments. Structural aberrations include chromosome fusions (such as end to end fusions, Robertsonian fusion like configurations).

### Bone Marrow Colony Forming Assay

BM cells (1×10^5^) seeded on 35 mm dishes in Methocult GF M3434 media (Stemcell Technologies Inc.) were either left untreated or treated with MMC (40 ng/ml) and cultured for 12 days prior to counting.

### Cell Death Assays

Cell death was assessed using 7AAD, PI or Annexin V-PI staining and FACS analysis as described [12].

### Immunobloting

Activated B-cells treated with 0.1 µg MMC (Sigma) were harvested and lysed 18 hr post-treatment. Proteins were detected using the following anti-rabbit antibodies: anti-p53 (FL393, Santa Cruz), anti-Phospho-p53 Ser-15 (cell signaling), anti-p21 (Santa cruz), anti-Bax (Santa cruz), anti-Survivin (Novus) and anti-β-actin (Sigma).

### Immunostaining

Passage 1 MEFs derived from *WT* and *Mus81^Δex3-4/Δex3-4^*, *Chk2^−/−^*, and *Mus81^Δex3-4/Δex3-4^Chk2^−/−^* embryos were seeded onto coverslips and either left untreated or treated with MMC (1 µg/ml). 24 hr post treatment, MEFs were fixed with 2% paraformaldehyde, blocked with antibody dilution buffer and stained with DAPI. Images were taken on a microscope (DM400B; Leica) under 100X magnification.

### Bone Marrow Transplantation

Bone marrow cells were harvested from the femur of 8 week old mice. 1×10^6^ cells were transplanted to 6 week old *Rag1^−/−^* mice by tail vein injection. The transplanted mice were scarified and analysed 8 weeks post-transplantation.

### *In Vivo* Sensitivity to MMC–Induced Damage

Mice (n = 10) for each genotype were injected (i.p) with 12.5 mg MMC/Kg of body mass and observed for two weeks post treatment. Mice were sacrificed when they became moribund and the day of sacrifice was counted as day of death.

### Statistical Analysis

The two tailed unpaired student's t test was used for statistical analysis except for survival curves where Log Rank test was employed (Prism 5, GraphPad Software).

### Ethics Statement

All experiments were performed in compliance with Ontario Cancer Institute animal care committee guidelines.

## Supporting Information

Figure S1 Weight of *Mus81^-/-^Chk2^-/-^* Females and Controls. Weight of 6 to 8 week old or 4 to 8 month old *WT*, *Mus81^Δex3-4/Δex3-4^*, *Chk2^-/-^*, and *Mus81^Δex3-4/Δex3-4^Chk2^-/-^* females. No difference was observed. *Mus81^-/-^*: *Mus81^Δex3-4/Δex3-4^*.
(0.55 MB TIF)

Figure S2 Effects of Chk2 Inactivation on Homeostasis of Lymphocytes from *Mus81^-/-^* Mice. (A) Ratio of Thy1.2^+^ and B220^+^ splenocytes from the 6 to 8 week old indicated mice. At least four independent experiments using one mouse per group were performed. (B) Total number of macrophages in spleen of the indicated mice. (C) Total number of CD4^−^CD8^−^ thymocytes in the indicated mice. (D) Total number of CD4^+^CD8^+^ thymocytes in the indicated mice. (E) Total number of CD4^+^ thymocytes in the indicated mice. (F) Total number of CD8^+^ thymocytes in the indicated mice. **: *P*  =  0.0002. *: *P* = 0.0001. At least four independent experiments using one mouse per group were performed. Bar graphs show means ± SEM. *Mus81^-/-^*: *Mus81^Δex3-4/Δex3-4^*.
(1.08 MB TIF)

Figure S3 Effects of Chk2 Inactivation on Death of Lymphocytes from *Mus81^-/-^* Mice. (A) Percentage of cell death of Naïve CD4^+^ T-cells from Spleen of *Mus81^Δex3-4/Δex3-4^Chk2^-/-^* Mice. (B) Percentage of cell death of Naïve CD8^+^ T-cells from Spleen of *Mus81^Δex3-4/Δex3-4^Chk2^-/-^* Mice. (C) Representative FACS analysis of cell death performed on untreated (UT) or LPS activated B-cells (48hr) from the indicated mice using PI assay. Numbers indicates the percent of dead cells. At least four independent experiments using one mouse per group were performed. Bar graphs show means ± SEM. *: *P*<0.04. *Mus81^-/-^*: *Mus81^Δex3-4/Δex3-4^*.
(4.35 MB TIF)

Figure S4 *Mus81^-/-^Chk2^-/-^* Mice Display Normal TCR Expression. (A) Representative FACS histograms of pan TCRVβ expression in thymocytes from *WT, Mus81^Δex3-4/Δex3-4^, Chk2^-/-^ and Mus81^Δex3-4/Δex3-4^Chk2^-/-^*mice. Bars indicate the TCRVβ^High^ positive cells. (B) Percentage of the TCRVβ (4, 5.1-5.2 and 17a) positive thymocytes. Numbers indicates the percent of TCRVβ positive cells. At least five independent experiments using one mouse per group were performed. Bar graphs show means ± SEM. (C) Representative FACS histograms of pan TCRVβ expression in splenocytes from *WT, Mus81^Δex3-4/Δex3-4^, Chk2^-/-^ and Mus81^Δex3-4/Δex3-4^Chk2^-/-^*mice. Bars indicate the TCRVβ positive cells. *Mus81^-/-^*: *Mus81^Δex3-4/Δex3-4^*.
(1.90 MB TIF)

Figure S5 Defective Homeostasis of the B-Cell Lineage in the *Mus81^-/-^Chk2^-/-^* Mice. Representative FACS analysis of anti-B220 and anti-IgM staining of the B220^+^CD43^−^ gated BM cells from *WT*, *Mus81^Δex3-4/Δex3-4^*, *Chk2^-/-^*, and *Mus81^Δex3-4/Δex3-4^Chk2^-/-^* mice (top). Representative FACS analysis of anti-IgD and anti-IgM staining of the B220^+^ gated BM cells from *WT*, *Mus81^Δex3-4/Δex3-4^*, *Chk2^-/-^*, and *Mus81^Δex3-4/Δex3-4^Chk2^-/-^* mice (bottom). At least four independent experiments using one mouse per group were performed. *Mus81^-/-^*: *Mus81^Δex3-4/Δex3-4^*.
(2.77 MB TIF)

Figure S6 The Developmental Defect of *Mus81^-/-^ Chk2^-/-^* Lymphocytes Is Cell Autonomous. (A) Representative FACS analysis of anti-B220 and anti-CD43 staining of the BM cells from *Rag1^-/-^*mice reconstituted with BM cells from *WT*, *Mus81^Δex3-4/Δex3-4^*, *Chk2^-/-^*, or *Mus81^Δex3-4/Δex3-4^Chk2^-/-^* mice. (B) Absolute cell number of recirculating (CD43^−^B220^high^) BM cells from *Rag1^-/-^* reconstituted mice as in (A). (C) Absolute number of total, CD4^+^ and CD8^+^ thymocytes of *Rag1^-/-^* reconstituted mice as in (A). (D) Absolute number of total splenocytes, B-cells, T- cells, CD4^+^ T-cells and CD8^+^ T-cells from *Rag1^-/-^* reconstituted mice as in (A). Numbers of cells are indicated. At least four independent experiments using one mouse per group were performed. Bar graphs show means ± SEM. *: *P*<0.05. *Mus81^-/-^*: *Mus81^Δex3-4/Δex3-4^*.
(5.17 MB TIF)

Figure S7 Effect of Chk2 Inactivation on the Proliferation and DNA Damage Response of *Mus81^-/-^* Lymphocytes. (A) Representative cell cycle analysis of *WT*, *Mus81^Δex3-4/Δex3-4^*, *Chk2^-/-^*, and *Mus81^Δex3-4/Δex3-4^Chk2^-/-^* activated T-cells (anti-CD3 + IL2) 120h post stimulation. Cells were either untreated (UT) or treated with 0.5 μg/ml MMC for 18hr (MMC). Percentages of cells in G0/G1, S, and G2/M are shown. (B) Activated (anti-CD3 + IL2) T-cells from *WT*, *Mus81^Δex3-4/Δex3-4^*, *Chk2^-/-^*, and *Mus81^Δex3-4/Δex3-4^Chk2^-/-^* mice were exposed to IR (4 Gy) and 12hr later cell death was examined using FACS analysis 7-AAD. Data presented is normalized to UT cells and bar graphs show means ± SEM. At least three independent experiments using one mouse per group were performed. ns: not statistically significant. *Mus81^-/-^*: *Mus81^Δex3-4/Δex3-4^*.
(1.03 MB TIF)

Figure S8 Reduced Expression Level of Survivin in MMC Treated *Mus81^-/-^Chk2^-/-^* B-Cells. Western blot analysis of LPS activated *WT*, *Mus81^Δex3-4/Δex3-4^, Chk2^-/-^and Mus81^Δex3-4/Δex3-4^Chk2^-/-^* B-cells either untreated or MMC treated for 18hr and 24hr. The level of expression of survivin and β-actin proteins is shown. *Mus81^-/-^*: *Mus81^Δex3-4/Δex3-4^*.
(0.16 MB TIF)

Table S1 Litter sizes and frequency of double mutants from interbreeding of *Mus81^Δex3-4/Δex3-4^Chk2* ^-/-^ mice or compound heterozygotes.
(0.04 MB DOC)

Table S2 *Mus81^Δex3-4/Δex3-4^Chk2^-/-^* females were born at the expected Mendelian ratio.
(0.03 MB DOC)

Table S3 Spontaneous and MMC–induced chromosomal aberrations of activated *Mus81^Δex3-4/Δex3-4^Chk2* ^-/-^ B-cells.
(0.07 MB DOC)

Table S4 Spontaneous and MMC–induced chromosomal aberrations of activated *Mus81^Δex3-4/Δex3-4^Chk2* ^-/-^ T-cells.
(0.07 MB DOC)

Table S5 Spontaneous chromosomal aberrations of *Mus81^Δex3-4/Δex3-4^Chk2* ^-/-^ primary MEFs.
(0.05 MB DOC)
