# Supplementary figures and images for: Inactivation of Chk2 and Mus81 Leads to Impaired Lymphocytes Development, Reduced Genomic Instability, and Suppression of Cancer
Source: PLoS Genet. 2011 May 19;7(5):e1001385. doi: 10.1371/journal.pgen.1001385 (PMC3098187; doi:10.1371/journal.pgen.1001385)

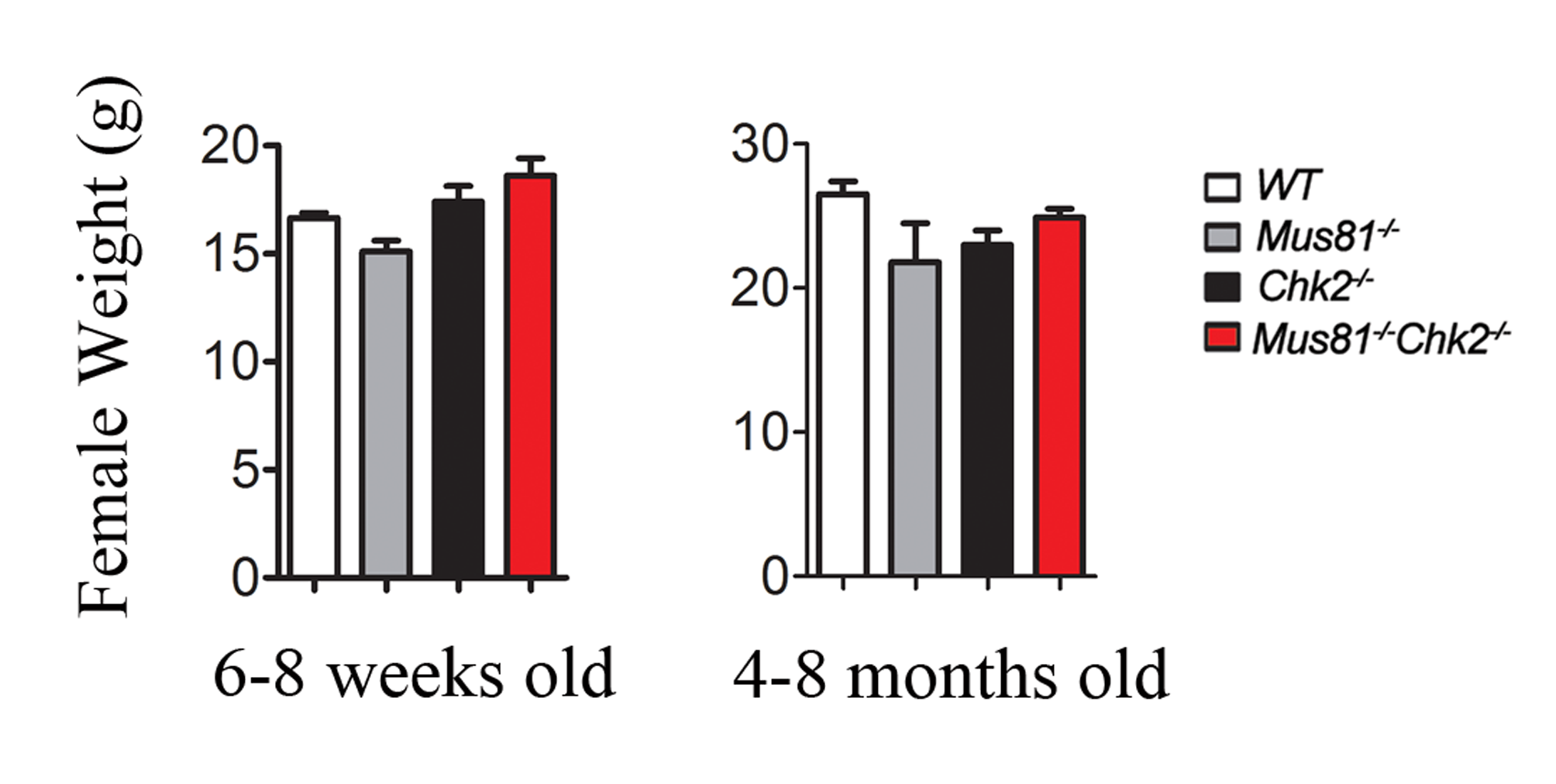

Supplement: Figure S1 — Weight of Mus81-/-Chk2-/- Females and Controls. Weight of 6 to 8 week old or 4 to 8 month old WT, Mus81Δex3-4/Δex3-4, Chk2-/-, and Mus81Δex3-4/Δex3-4Chk2-/- females. No difference was observed. Mus81-/-: Mus81Δex3-4/Δex3-4. (0.55 MB TIF) [file pgen.1001385.s001.tif]

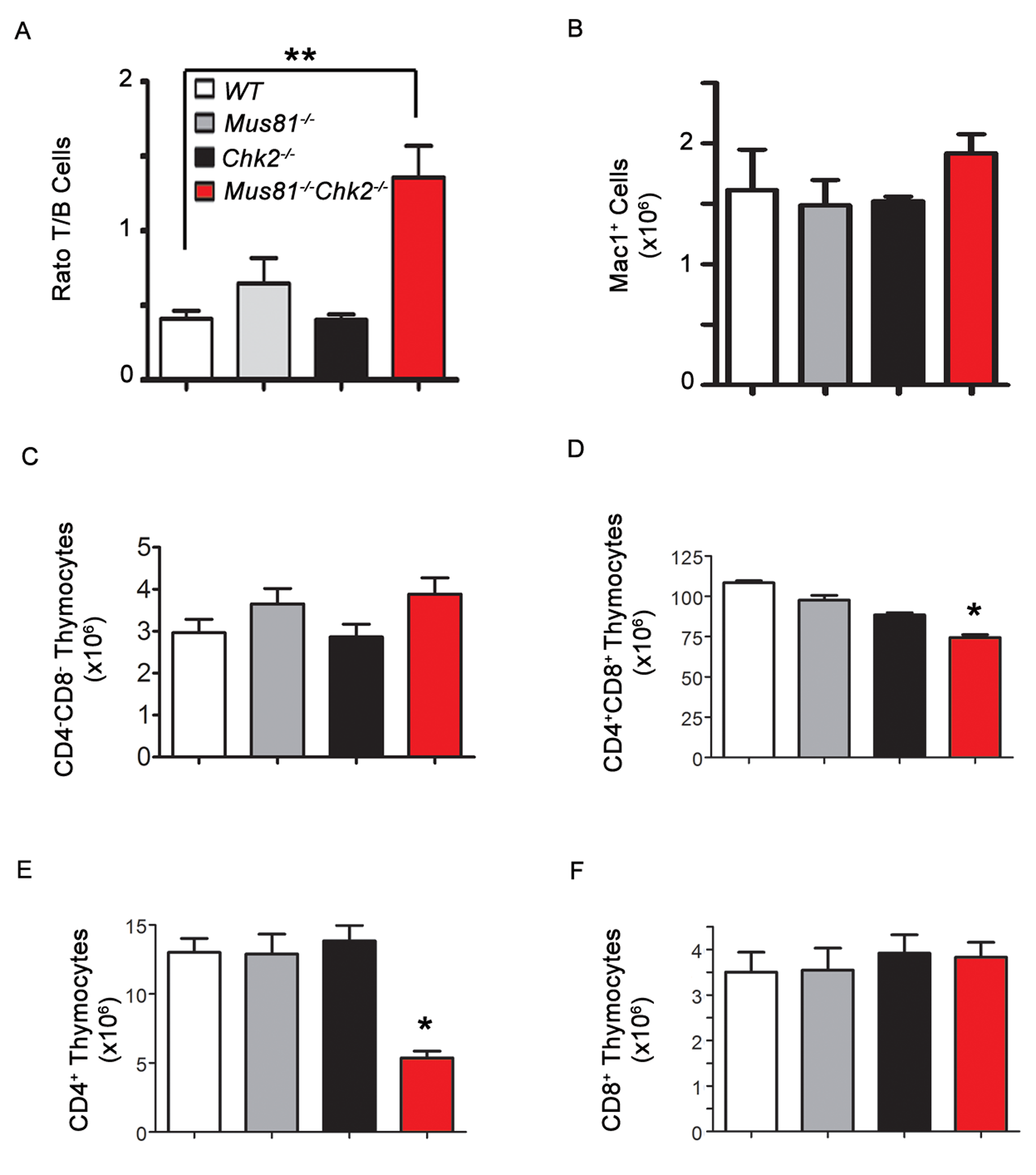

Supplement: Figure S2 — Effects of Chk2 Inactivation on Homeostasis of Lymphocytes from Mus81-/- Mice. (A) Ratio of Thy1.2+ and B220+ splenocytes from the 6 to 8 week old indicated mice. At least four independent experiments using one mouse per group were performed. (B) Total number of macrophages in spleen of the indicated mice. (C) Total number of CD4−CD8− thymocytes in the indicated mice. (D) Total number of CD4+CD8+ thymocytes in the indicated mice. (E) Total number of CD4+ thymocytes in the indicated mice. (F) Total number of CD8+ thymocytes in the indicated mice. **: P = 0.0002. *: P = 0.0001. At least four independent experiments using one mouse per group were performed. Bar graphs show means ± SEM. Mus81-/-: Mus81Δex3-4/Δex3-4. (1.08 MB TIF) [file pgen.1001385.s002.tif]

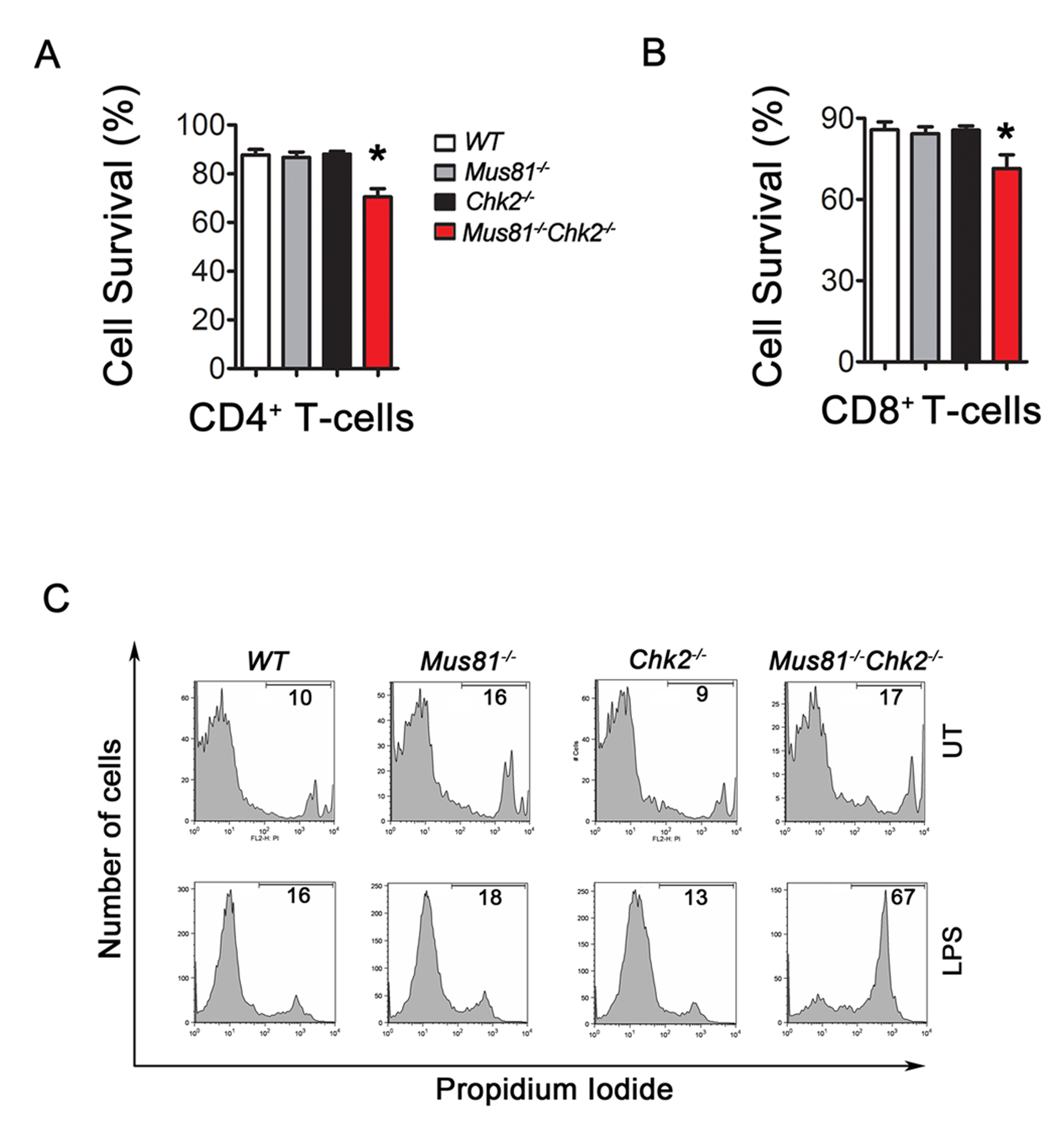

Supplement: Figure S3 — Effects of Chk2 Inactivation on Death of Lymphocytes from Mus81-/- Mice. (A) Percentage of cell death of Naïve CD4+ T-cells from Spleen of Mus81Δex3-4/Δex3-4Chk2-/- Mice. (B) Percentage of cell death of Naïve CD8+ T-cells from Spleen of Mus81Δex3-4/Δex3-4Chk2-/- Mice. (C) Representative FACS analysis of cell death performed on untreated (UT) or LPS activated B-cells (48hr) from the indicated mice using PI assay. Numbers indicates the percent of dead cells. At least four independent experiments using one mouse per group were performed. Bar graphs show means ± SEM. *: P<0.04. Mus81-/-: Mus81Δex3-4/Δex3-4. (4.35 MB TIF) [file pgen.1001385.s003.tif]

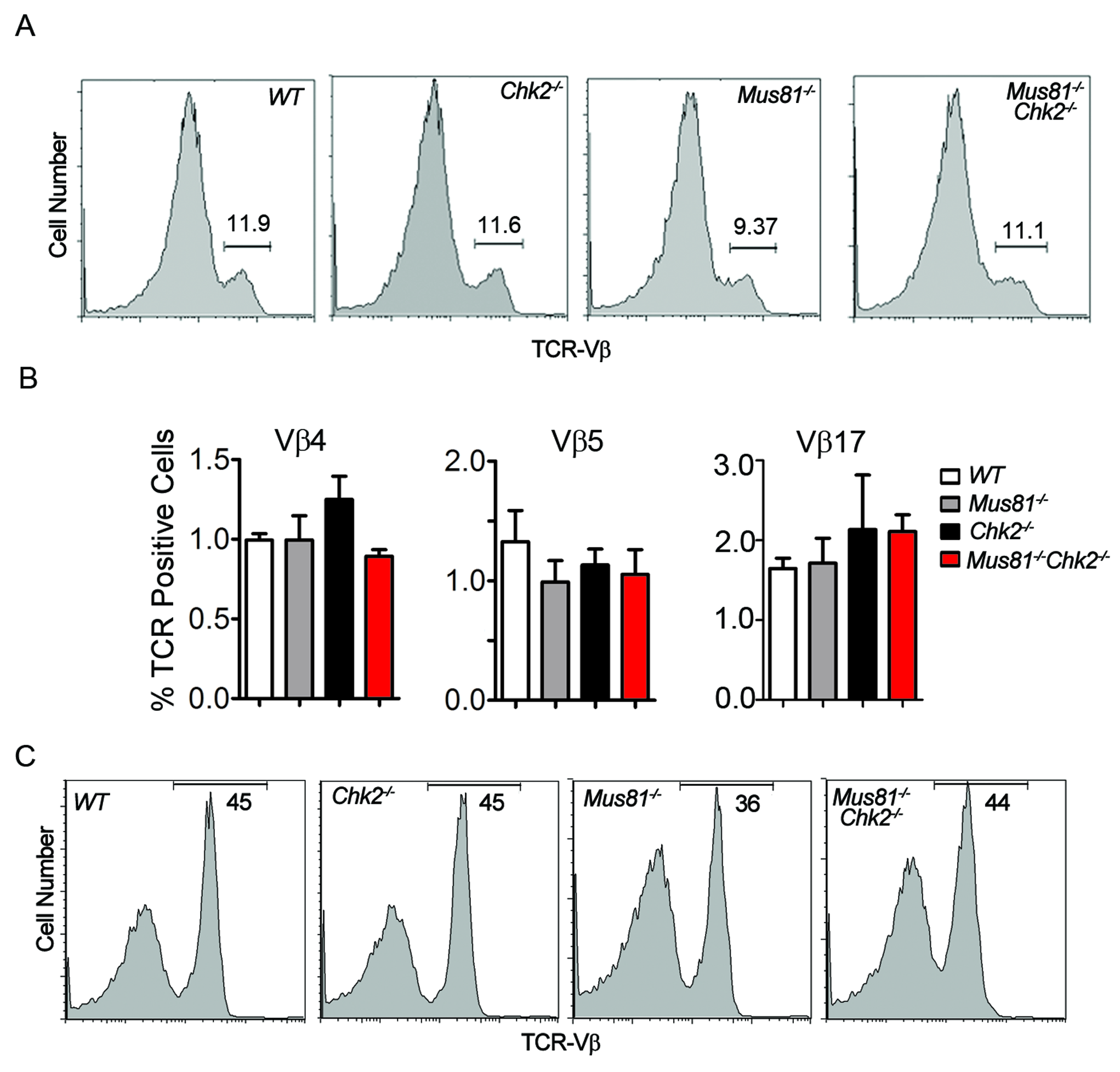

Supplement: Figure S4 — Mus81-/-Chk2-/- Mice Display Normal TCR Expression. (A) Representative FACS histograms of pan TCRVβ expression in thymocytes from WT, Mus81Δex3-4/Δex3-4, Chk2-/- and Mus81Δex3-4/Δex3-4Chk2-/-mice. Bars indicate the TCRVβHigh positive cells. (B) Percentage of the TCRVβ (4, 5.1-5.2 and 17a) positive thymocytes. Numbers indicates the percent of TCRVβ positive cells. At least five independent experiments using one mouse per group were performed. Bar graphs show means ± SEM. (C) Representative FACS histograms of pan TCRVβ expression in splenocytes from WT, Mus81Δex3-4/Δex3-4, Chk2-/- and Mus81Δex3-4/Δex3-4Chk2-/-mice. Bars indicate the TCRVβ positive cells. Mus81-/-: Mus81Δex3-4/Δex3-4. (1.90 MB TIF) [file pgen.1001385.s004.tif]

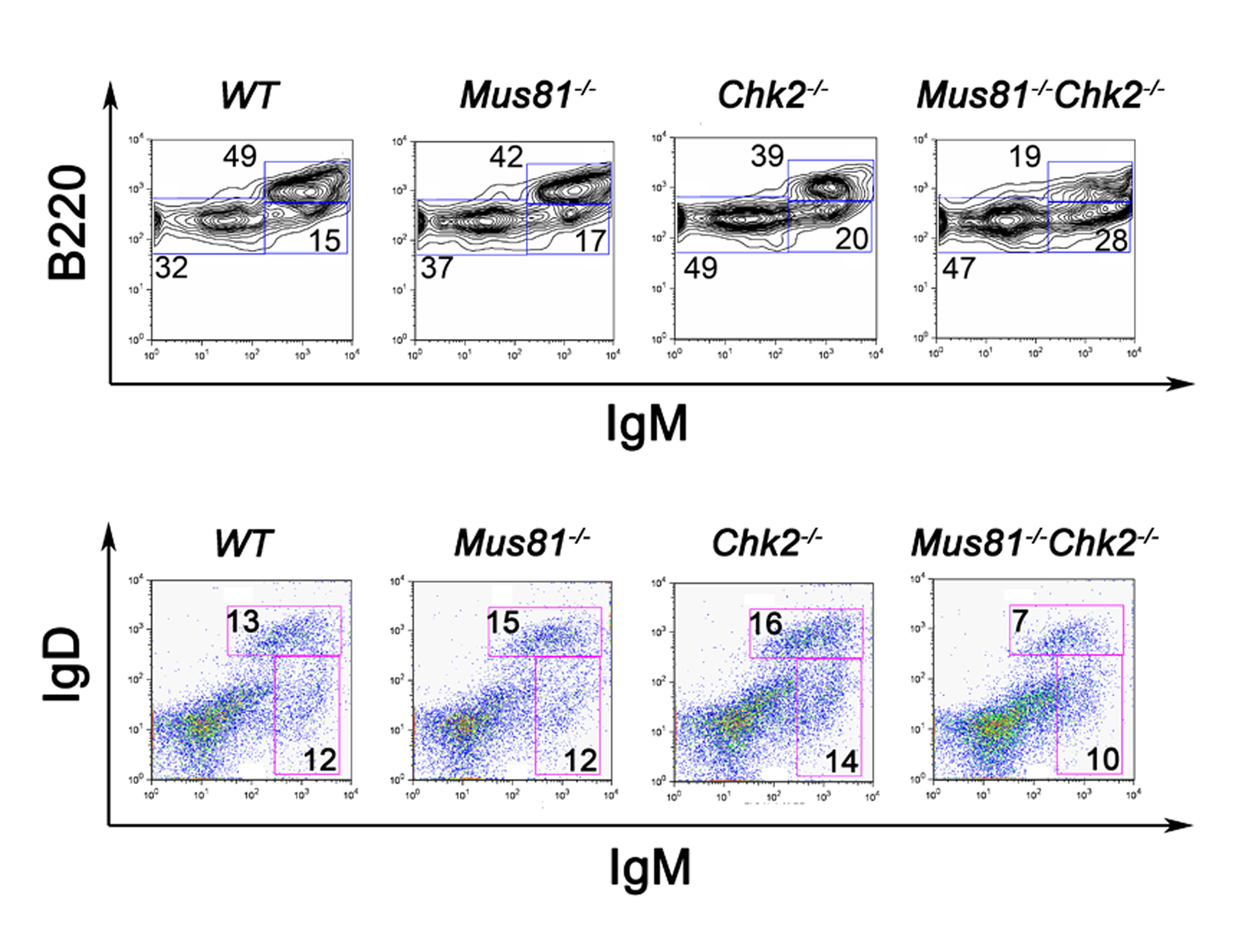

Supplement: Figure S5 — Defective Homeostasis of the B-Cell Lineage in the Mus81-/-Chk2-/- Mice. Representative FACS analysis of anti-B220 and anti-IgM staining of the B220+CD43− gated BM cells from WT, Mus81Δex3-4/Δex3-4, Chk2-/-, and Mus81Δex3-4/Δex3-4Chk2-/- mice (top). Representative FACS analysis of anti-IgD and anti-IgM staining of the B220+ gated BM cells from WT, Mus81Δex3-4/Δex3-4, Chk2-/-, and Mus81Δex3-4/Δex3-4Chk2-/- mice (bottom). At least four independent experiments using one mouse per group were performed. Mus81-/-: Mus81Δex3-4/Δex3-4. (2.77 MB TIF) [file pgen.1001385.s005.tif]

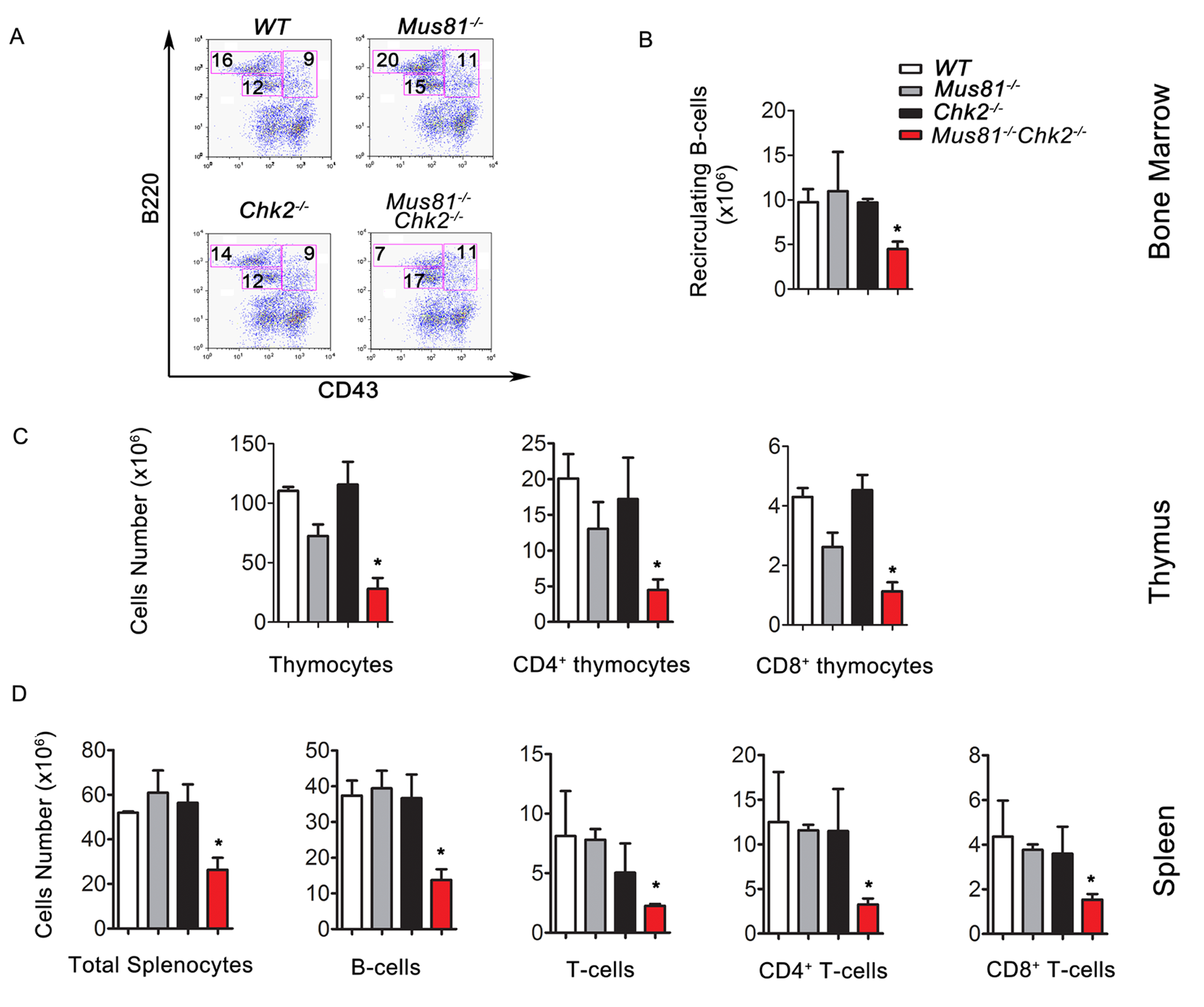

Supplement: Figure S6 — The Developmental Defect of Mus81-/- Chk2-/- Lymphocytes Is Cell Autonomous. (A) Representative FACS analysis of anti-B220 and anti-CD43 staining of the BM cells from Rag1-/-mice reconstituted with BM cells from WT, Mus81Δex3-4/Δex3-4, Chk2-/-, or Mus81Δex3-4/Δex3-4Chk2-/- mice. (B) Absolute cell number of recirculating (CD43−B220high) BM cells from Rag1-/- reconstituted mice as in (A). (C) Absolute number of total, CD4+ and CD8+ thymocytes of Rag1-/- reconstituted mice as in (A). (D) Absolute number of total splenocytes, B-cells, T- cells, CD4+ T-cells and CD8+ T-cells from Rag1-/- reconstituted mice as in (A). Numbers of cells are indicated. At least four independent experiments using one mouse per group were performed. Bar graphs show means ± SEM. *: P<0.05. Mus81-/-: Mus81Δex3-4/Δex3-4. (5.17 MB TIF) [file pgen.1001385.s006.tif]

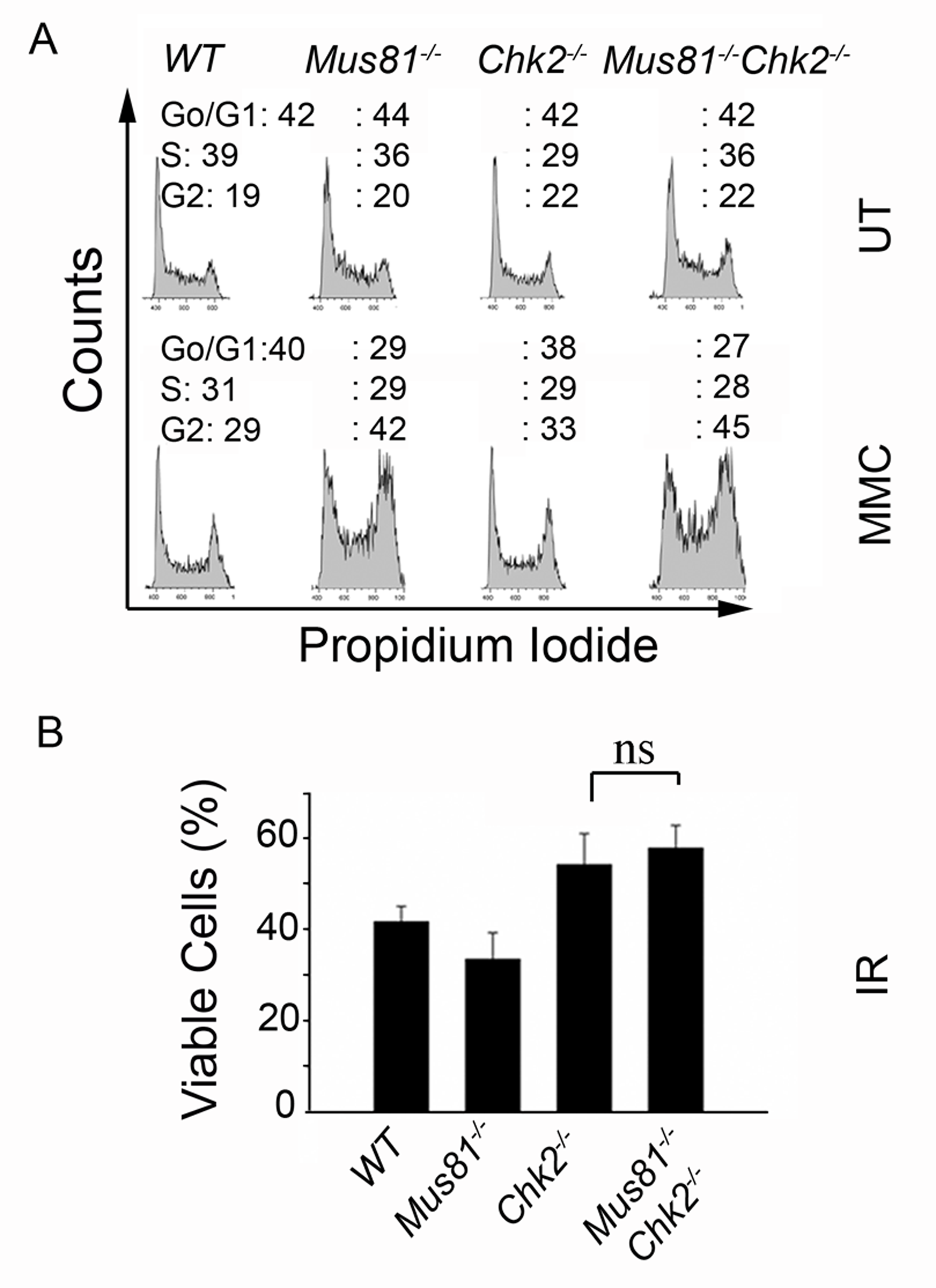

Supplement: Figure S7 — Effect of Chk2 Inactivation on the Proliferation and DNA Damage Response of Mus81-/- Lymphocytes. (A) Representative cell cycle analysis of WT, Mus81Δex3-4/Δex3-4, Chk2-/-, and Mus81Δex3-4/Δex3-4Chk2-/- activated T-cells (anti-CD3 + IL2) 120h post stimulation. Cells were either untreated (UT) or treated with 0.5 μg/ml MMC for 18hr (MMC). Percentages of cells in G0/G1, S, and G2/M are shown. (B) Activated (anti-CD3 + IL2) T-cells from WT, Mus81Δex3-4/Δex3-4, Chk2-/-, and Mus81Δex3-4/Δex3-4Chk2-/- mice were exposed to IR (4 Gy) and 12hr later cell death was examined using FACS analysis 7-AAD. Data presented is normalized to UT cells and bar graphs show means ± SEM. At least three independent experiments using one mouse per group were performed. ns: not statistically significant. Mus81-/-: Mus81Δex3-4/Δex3-4. (1.03 MB TIF) [file pgen.1001385.s007.tif]

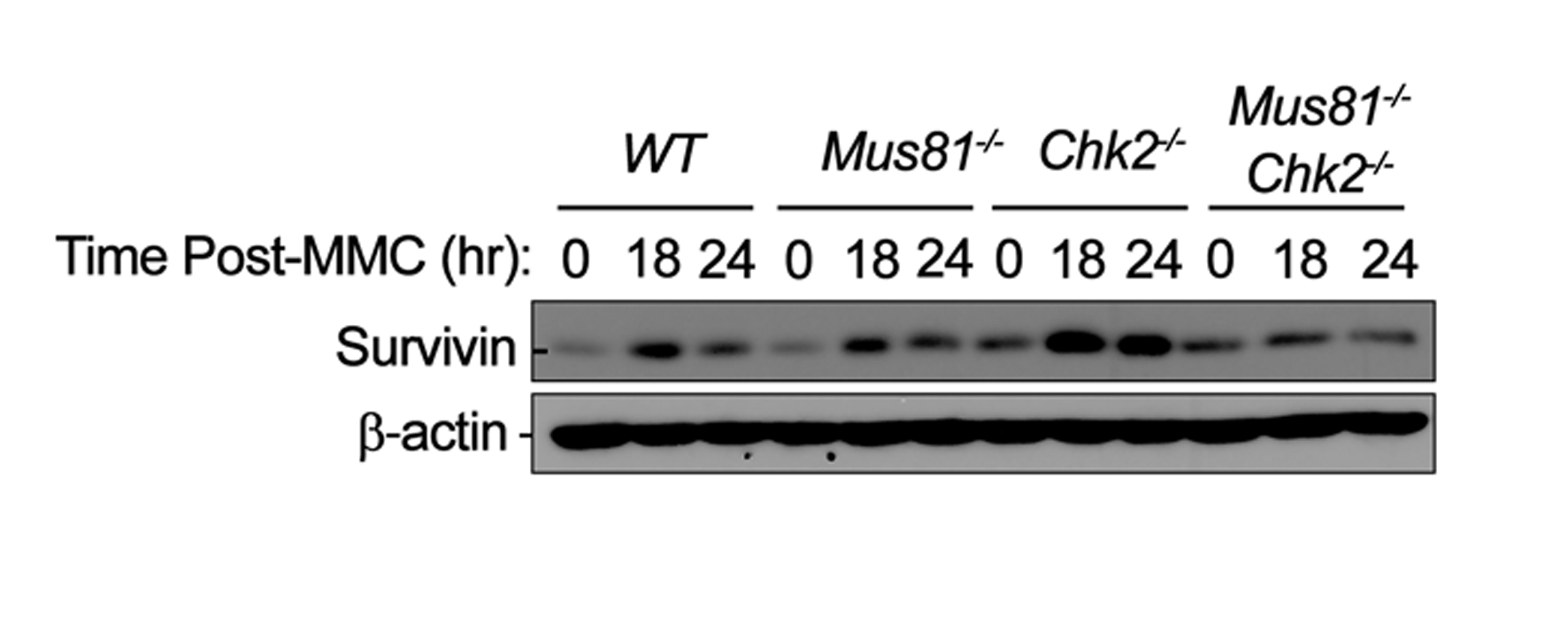

Supplement: Figure S8 — Reduced Expression Level of Survivin in MMC Treated Mus81-/-Chk2-/- B-Cells. Western blot analysis of LPS activated WT, Mus81Δex3-4/Δex3-4, Chk2-/-and Mus81Δex3-4/Δex3-4Chk2-/- B-cells either untreated or MMC treated for 18hr and 24hr. The level of expression of survivin and β-actin proteins is shown. Mus81-/-: Mus81Δex3-4/Δex3-4. (0.16 MB TIF) [file pgen.1001385.s008.tif]
