# Supplementary material for: Inactivation of Chk2 and Mus81 Leads to Impaired Lymphocytes Development, Reduced Genomic Instability, and Suppression of Cancer
Source: PLoS Genet. 2011 May 19;7(5):e1001385. doi: 10.1371/journal.pgen.1001385 (PMC3098187; doi:10.1371/journal.pgen.1001385)
Supplement: Table S1 — Litter sizes and frequency of double mutants from interbreeding of Mus81Δex3-4/Δex3-4Chk2 -/- mice or compound heterozygotes. (0.04 MB DOC) [file pgen.1001385.s009.doc]

**Table S1**: Litter sizes and frequency of double mutants from interbreeding of *Mus81^Δex3-4/Δex3-4^Chk2*^-/-^ mice or compound heterozygotes.

| **Breeding pairs** | **Litter size (pups)** | **Genotypes** |
| --- | --- | --- |
|  | | |
| *Chk2^-/-^* x *Chk2^-/-^* | 8.0 ± 1.3 | Expected % *Chk2^-/-^* mice: 100%  Obtained % *Chk2^-/-^* mice: 100% |
| *Mus81^-/-^* x *Mus81^-/-^* | 7.1 ± 0.7 | Expected % *Mus81^-/-^*: 100%  Obtained % *Mus81^-/-^*: 100% |
| *Mus81^+/-^Chk2^+/-^* x *Mus81^+/-^Chk2^+/-^* | 9.3 ± 1 | Expected % *Mus81^-/-^Chk2^-/-^* mice: 6.25%  Obtained % *Mus81^-/-^Chk2^-/-^* mice: 7.14% |
| *Mus81^-/-^Chk2^-/-^* x *Mus81^-/-^Chk2^+/-^* | 8.5 ± 0.5 | Expected % *Mus81^-/-^Chk2^-/-^* mice: 50%  Obtained % *Mus81^-/-^Chk2^-/-^* mice: 42% |
| *Mus81^-/-^Chk2^-/-^* x *Mus81^-/-^Chk2^-/-^* | 8.3 ± 0.4 | Expected % *Mus81^-/-^Chk2^-/-^* mice: 100%  Obtained % *Mus81^-/-^Chk2^-/-^* mice: 100% |
