# Supplementary material for: Inactivation of Chk2 and Mus81 Leads to Impaired Lymphocytes Development, Reduced Genomic Instability, and Suppression of Cancer
Source: PLoS Genet. 2011 May 19;7(5):e1001385. doi: 10.1371/journal.pgen.1001385 (PMC3098187; doi:10.1371/journal.pgen.1001385)
Supplement: Table S2 — Mus81Δex3-4/Δex3-4Chk2-/- females were born at the expected Mendelian ratio. (0.03 MB DOC) [file pgen.1001385.s010.doc]

**Table S2:** *Mus81^Δex3-4/Δex3-4^Chk2^-/-^* females were born at the expected Mendelian ratio.

| Breeding pairs | Observed males (%) | Observed females (%) | *P* value |
| --- | --- | --- | --- |
|  | | | |
| *Chk2^-/-^* x *Chk2^-/-^* | 55% ± 11 | 45% ± 11 | < 0.05 |
| *Mus81^-/-^* x *Mus81^-/-^* | 56.9% ± 6 | 43% ± 6 | < 0.05 |
| *Mus81^-/-^Chk2^-/-^* x *Mus81^-/-^Chk2^-/-^* | 43.6% ± 6 | 56.4 ± 6 | < 0.05 |
