# Supplementary material for: Inactivation of Chk2 and Mus81 Leads to Impaired Lymphocytes Development, Reduced Genomic Instability, and Suppression of Cancer
Source: PLoS Genet. 2011 May 19;7(5):e1001385. doi: 10.1371/journal.pgen.1001385 (PMC3098187; doi:10.1371/journal.pgen.1001385)
Supplement: Table S3 — Spontaneous and MMC–induced chromosomal aberrations of activated Mus81Δex3-4/Δex3-4Chk2 -/- B-cells. (0.07 MB DOC) [file pgen.1001385.s011.doc]

**Table S3: Spontaneous and MMC induced chromosomal aberrations of activated *Mus81^Δex3-4/Δex3-4^Chk2*^-/-^ B-cells**

| **Sample ID** | **Metaphases** | **Aneuploid** | **Aberrant** | **Fragments/** | **Fusions** | **Triradial-like** | **Total** |
| --- | --- | --- | --- | --- | --- | --- | --- |
|  | **Scored** | **Cells** | **Cells** | **Breaks** |  | **Structures** | **Aberrations** |
|  |  |  |  |  |  |  |  |
| *WT* | 120 | 0 | 1 | 1 | 0 | 0 | 1 |
|  |  | **0** | **0.8** | **0.8** | **0** | **0** | **0.8** |
|  |  |  |  |  |  |  |  |
| *WT* (MMC) | 122 | 18 | 10 | 10 | 2 | 0 | 12 |
|  |  | **14.7** | **8.1** | **8.1** | **1.6** | **0** | **9.8** |
|  |  |  |  |  |  |  |  |
| *Mus81*^-/-^ | 109 | 7 | 10 | 11 | 0 | 0 | 11 |
|  |  | **6.4** | **9.1** | **10** | **0** | **0** | **10** |
|  |  |  |  |  |  |  |  |
| *Mus81^-/-^* (MMC) | 109 | 17 | 29 | 30 | 2 | 2 | 34 |
|  |  | **15.5** | **26.6** | **27.5** | **1.8** | **1.8** | **31.1** |
|  |  |  |  |  |  |  |  |
| *Chk2^-/-^* | 107 | 4 | 3 | 3 | 0 | 0 | 3 |
|  |  | **3.7** | **2.8** | **2.8** | **0** | **0** | **2.8** |
|  |  |  |  |  |  |  |  |
| *Chk2^-/-^* (MMC) | 108 | 13 | 16 | 15 | 2 | 0 | 17 |
|  |  | **12** | **14.8** | **13.8** | **1.85** | **0** | **15.7** |
|  |  |  |  |  |  |  |  |
| *Mus81^-/-^Chk2^-/-^* | 125 | 12 | 3 | 3 | 0 | 0 | 3 |
|  |  | **9.6** | **2.4** | **2.4** | **0** | **0** | **2.4** |
| *Mus81^-/-^Chk2^-/-^* (MMC) | 134 | 38 | 51 | 67 | 0 | 7 | 74 |
|  |  | **28.3** | **38** | **50** | **0** | **5.2** | **55** |
