# Supplementary material for: Inactivation of Chk2 and Mus81 Leads to Impaired Lymphocytes Development, Reduced Genomic Instability, and Suppression of Cancer
Source: PLoS Genet. 2011 May 19;7(5):e1001385. doi: 10.1371/journal.pgen.1001385 (PMC3098187; doi:10.1371/journal.pgen.1001385)
Supplement: Table S4 — Spontaneous and MMC–induced chromosomal aberrations of activated Mus81Δex3-4/Δex3-4Chk2 -/- T-cells. (0.07 MB DOC) [file pgen.1001385.s012.doc]

**Table S4:** Spontaneous and MMC induced chromosomal aberrations of activated *Mus81^Δex3-4/Δex3-4^Chk2*^-/-^ T-cells

| **Sample ID** | **Metaphases** | **Aneuploid** | **Aberrant** | **Fragments/** | **Fusions** | **Triradial-like** | **Total** |
| --- | --- | --- | --- | --- | --- | --- | --- |
|  | **Scored** | **Cells** | **Cells** | **Breaks** |  | **Structures** | **Aberrations** |
|  |  |  |  |  |  |  |  |
| *WT* | 102 | 0 | 1 | 3 | 0 | 0 | 3 |
|  |  | **0.00** | **0.98** | **2.94** |  |  | **2.94** |
|  |  |  |  |  |  |  |  |
| *WT* (MMC) | 119 | 19 | 20 | 13 | 6 | 7 | 26 |
|  |  | **15.97** | **16.81** | **10.92** | **7.00** | **5.88** | **21.85** |
|  |  |  |  | (8 chromatid) |  |  |  |
| *Mus81*^-/-^ | 102 | 7 | 14 | 13 | 2 | 0 | 15 |
|  |  | **6.86** | **13.73** | **12.75** | **1.96** |  | **14.71** |
|  |  |  |  | (12 chromatid) |  |  |  |
| *Mus81^-/-^* (MMC) | 104 | 16 | 31 | 31 | 9 | 4 | 44 |
|  |  | **15.38** | **29.81** | **29.81** | **8.65** | **3.85** | **42.31** |
|  |  |  |  | (27 Chromatid) |  |  |  |
| *Chk2^-/-^* | 113 | 5 | 7 | 7 | 1 | 0 | 8 |
|  |  | **4.42** | **6.19** | **6.19** | **0.88** |  | **7.08** |
|  |  |  |  | (7 chromatid) |  |  |  |
| *Chk2^-/-^* (MMC) | 118 | 15 | 25 | 14 | 6 | 8 | 28 |
|  |  | **12.71** | **21.19** | **11.86** | **5.08** | **6.78** | **23.73** |
|  |  |  |  | (10 Chromatid) |  |  |  |
| *Mus81^-/-^Chk2^-/-^* | 102 | 3 | 11 | 9 | 0 | 2 | 11 |
|  |  | **2.94** | **10.78** | **8.82** |  | **1.96** | **10.78** |
|  |  |  |  | **(**6 chromatid**)** |  |  |  |
| *Mus81^-/-^Chk2^-/-^* (MMC) | 107 | 37 | 64 | 63 | 17 | 11 | 91 |
|  |  | **34.58** | **59.81** | **58.88** | **15.89** | **10.28** | **85.05** |
|  |  |  |  | (41 chromatid) |  |  |  |
