# Supplementary material for: Inactivation of Chk2 and Mus81 Leads to Impaired Lymphocytes Development, Reduced Genomic Instability, and Suppression of Cancer
Source: PLoS Genet. 2011 May 19;7(5):e1001385. doi: 10.1371/journal.pgen.1001385 (PMC3098187; doi:10.1371/journal.pgen.1001385)
Supplement: Table S5 — Spontaneous chromosomal aberrations of Mus81Δex3-4/Δex3-4Chk2 -/- primary MEFs. (0.05 MB DOC) [file pgen.1001385.s013.doc]

**Table S5: Spontaneous chromosomal aberrations of *Mus81^Δex3-4/Δex3-4^Chk2*^-/-^ primary MEFs.**

| **Sample ID** | **Metaphases** | **Aneuploid** | **Aberrant** | **Fragments/** | **Fusions** | **Triradial-like** | **Total** |
| --- | --- | --- | --- | --- | --- | --- | --- |
|  | **Scored** | **Cells** | **Cells** | **Breaks** |  | **Structures** | **Aberrations** |
|  |  |  |  |  |  |  |  |
|  |  |  |  |  |  |  |  |
| *WT* | 65 | 0 | 3 | 3 | 0 | 0 | 3 |
|  |  | **0.00** | **4.62** | **4.62** | **0.00** | **0.00** | **4.62** |
|  |  |  |  |  |  |  |  |
|  |  |  |  |  |  |  |  |
| *Mus81^-/-^* | 52 | 4 | 8 | 10 | 1 | 1 | **12** |
|  |  | **7.69** | **15.38** | **19.23** | **1.92** |  | **23.08** |
|  |  |  |  |  |  |  |  |
|  |  |  |  |  |  |  |  |
| *Chk2^-/-^* | 55 | 1 | 6 | 7 | 0 | 1 | **8** |
|  |  | **1.82** | **10.91** | **12.73** | **0.00** | **1.82** | **14.55** |
|  |  |  |  |  |  |  |  |
|  |  |  |  |  |  |  |  |
| *Mus81^-/-^ Chk2^-/-^* | 65 | 2 | 8 | 7 | 1 | 1 | **9** |
|  |  | **3.08** | **12.31** | **10.77** | **1.54** | **1.54** | **13.85** |
|  |  |  |  |  |  |  |  |
